# Glazed and unglazed transpired solar collectors for sustainable buildings: a comparative assessment

**DOI:** 10.1038/s41598-026-56698-w

**Published:** 2026-06-06

**Authors:** Mohammad Fawaier, Nabeel Abu Shaban, Laith Al-Hyari, Ahmad Al Aboushi, Omar Alharasees, Haitham A. Al Hasanat

**Affiliations:** 1 Department of Aviation Sciences and Management, Faculty of Aviation Sciences, Amman Arab University, Amman, Jordan; 2 Department of Mechanical Engineering, Faculty of Engineering and Technology, Al-Zaytoonah University, P.O. Box 130, Amman, 11733 Jordan; 3 Department of Building Service Engineering and Process Engineering, Faculty of Mechanical Engineering, Budapest University of Technology and Economics, Muegyetem rkp. 3-9, Budapest, H-1111 Hungary; 4 Department of Civil Engineering, Faculty of Engineering, Jerash University, Jerash, 26150 Jordan; 5 Department of Civil Engineering, College of Engineering, Amman Arab University, Amman, Jordan

**Keywords:** Dynamic insulation, Energy-saving, Thermal insulation, NZEB, Building physics, Energy science and technology, Engineering, Environmental sciences

## Abstract

Transpired solar collectors (TSCs) preheat ventilation air and reduce conductive heat loss through the building envelope, thereby acting as dynamic insulation. Direct outdoor comparisons of glazed and unglazed TSC modules operating under identical conditions remain limited. A systematic experimental study was conducted on two façade-mounted modules with the same perforated galvanised-steel absorber and 110 mm plenum: an unglazed TSC (UTSC) and a glazed TSC (GTSC) fitted with 4 mm low-iron tempered glass. The system was tested outdoors in Amman, Jordan, under clear-sky conditions (January-March 2025) at specific airflow rates of 18–144 m^3^/(h·m^2^) and solar irradiance of 200–1000 W/m^2^. Passive natural-convection operation of the GTSC was also evaluated at several cavity heights. Performance was quantified using physically bounded metrics covering thermal performance, heat exchange, exergy, wall heat-loss recapture, ventilation load reduction, and economic return. Across the tested range, the GTSC achieved thermal efficiency of 48–75% and exergy efficiency up to 12%, outperforming the UTSC (42–65%) by 6–9% points. The UTSC wall heat-loss recapture index decreased from 93% at low irradiance to 63% at peak irradiance, consistent with dynamic-insulation behaviour, while GTSC ventilation load reduction increased monotonically from 60% to 90%. Under passive operation, the GTSC reached 55% efficiency at 3.0 m cavity height without fan energy. Economic assessment using Jordanian energy prices indicated payback periods of 4.4 years (UTSC) and 5.2 years (GTSC). The results provide experimentally grounded guidance on when glazing is justified for sustainable ventilation preheating in buildings.

## Introduction

Energy use in buildings has increased in recent decades, mainly due to urban growth and higher expectations for indoor comfort^1^. This trend has placed more emphasis on practical measures that improve efficiency while using renewable resources^2^. Among the available options, the use of solar radiation for space heating and ventilation has received attention because it reduces reliance on conventional fuels and supports lower operational energy use^3–5^. Air-based solar thermal systems have been studied widely for this purpose, as they can preheat ventilation air directly and can be integrated into building façades. This approach also supports better use of the building envelope, with straightforward integration into ventilation pathways and reduced dependence on conventional heating during sunny periods^6,7^.

Buildings are responsible for roughly 40% of global primary energy consumption, and space heating dominates the demand in cold and transitional climates^8,9^. This reality has spurred widespread interest in passive and semi-passive solar systems that can reduce heating loads without large capital investments or complex maintenance requirements.

Transpired solar collectors (TSCs) occupy an appealing niche within this landscape: a thin, dark-coated, perforated metal sheet mounted a short distance in front of a south-facing wall absorbs solar radiation, heats the boundary-layer air on its outer surface, and draws that pre-warmed air through the perforations into a plenum by means of a low-power fan or natural buoyancy. The result is a system that simultaneously pre-heats outdoor ventilation supply air and creates a warm air film that reduces conductive heat loss through the underlying wall-effectively acting as an active or dynamic insulation layer^10–12^.

The heat-transfer fundamentals of unglazed TSCs were established in a series of landmark studies in the 1990s. Kutscher and colleagues^13,14^ developed analytical models for convective and radiative losses from the absorber plate and derived the correlation between plenum depth, perforation geometry and heat exchange effectiveness. Van Decker et al.^15^ extended these correlations to circular perforations on square and triangular pitch patterns, providing the basis for engineering design of arbitrary hole arrangements.

Leon and Kumar^16^ used these foundations to develop a complete mathematical framework for predicting UTSC performance across a wide range of climatic and operational parameters. More recently, attention has shifted toward evaluating TSCs in real building applications^17,18^, quantifying their economic and environmental benefits^19^, and incorporating them into nearly zero-energy building (NZEB) strategies^20^. A persistent limitation of conventional unglazed TSCs is their sensitivity to adverse ambient conditions. At wind speeds above roughly 2 m/s, convective heat loss from the exposed absorber surface increases substantially, eroding efficiency^21^. Under low-irradiance or overcast sky conditions, long-wave radiative emission from the hot black absorber to the cold sky becomes a significant competing loss pathway^22^.

Glazed TSCs (GTSCs) address both mechanisms by enclosing the absorber within a transparent cover: the air gap between glass and absorber suppresses external convective exposure, and the glass acts as a long-wave radiative trap. Gao et al.^23^ demonstrated a 20% thermal efficiency improvement over an unglazed reference by introducing a glazed perforated corrugated-plate design. Further, work by the same group^24^ confirmed that a glazed solar preheating collector wall can achieve meaningful ventilation load reductions across an entire heating season. Mahmood^25^ reported outdoor thermal efficiencies of 48–75% for a glazed flat-plate solar air heater under semi-arid conditions comparable to those of the present study.

Despite these encouraging results, the literature lacks direct experimental comparisons between UTSC and GTSC modules that share identical absorber geometry and are tested under the same outdoor conditions with the same instrumentation. Nearly all published comparisons either test configurations with different perforation patterns, different plate dimensions or different operating environments, making it impossible to isolate the specific contribution of glazing from other design variables. This is the gap the present study addresses.

Two TSC modules were fabricated with identical 3.0 m x 2.0 m galvanised-steel absorbers: one unglazed and one with a 4 mm low-iron glass cover installed 30 mm in front of the absorber. The modules were mounted side-by-side on a south-facing test façade at Al-Zaytoonah University, Amman, Jordan (31.83°N, 35.89°E) and tested simultaneously with the same instrumentation suite during clear-sky winter days in January-March 2025. Amman was chosen for its high solar radiation resource (annual daily irradiance on south-facing surfaces of 4–7 kWh/m²^26^, mild prevailing winds (1.0–1.9 m/s during the test months^27^, and cool winters that maximise the dynamic-insulation benefit of TSC operation.

The study evaluates thermal efficiency, heat exchange effectiveness, exergy efficiency, wall heat-loss recapture and ventilation load reduction using definitions that are physically bounded and internally consistent, addressing a further gap identified in the literature where some published metrics produce values exceeding their physical limits^28^. Natural-convection operation of the GTSC is also characterised over a range of cavity heights, providing design data for fully passive façade integration.

### Aim of this study

The specific objectives of the investigation are to:

- Design, fabricate and simultaneously test UTSC and GTSC modules sharing identical absorber geometry on the same south-facing façade, eliminating environmental and geometric confounds.
- Measure and compare thermal efficiency, heat exchange effectiveness, exergy efficiency, wall heat-loss recapture index (RI) and ventilation load reduction (VLR) over q_s = 18–144 m³/(h·m²) and G = 200–1000 W/m².
- Characterise passive natural-convection operation of the GTSC at cavity heights of 1.0–3.0 m.
- Validate a one-dimensional analytical model and derive design guidelines and an economic assessment for the Amman climate.

**Table 1:** Positioning of this study relative to selected prior work on transpired solar collectors.

Aspect	Typical prior work	This study	Contribution of this study
Experimental comparison	Single configuration only^13,16,21,23^	UTSC and GTSC side-by-side, same façade	Glazing effect isolated; no geometric or environmental confounds
Absorber geometry	Varied between studies^15,21,23^	Identical for both modules: 1 mm hole, 15 mm pitch, 110 mm plenum	Clean benchmark; geometry is not a confound
Airflow metric	Mass flow [kg/s] or un-normalised	q_s [m³/(h·m²)] throughout	Design-scalable; directly comparable across collector sizes
Metric physical bounds	Often implicit or violated^28^	RI, VLR each bounded in [0%, 100%]	Enables reproducible cross-study comparison
Natural convection	Tower geometries^29^	Compact wall-integrated GTSC, H = 1.0–3.0 m	First NC data for compact glazed TSC at building scale
Multi-parameter view	2D graphs only^16,21^	3D surfaces, contour maps	Reveals interaction effects; aids control strategy design
Economic analysis	Absent or partial^25^	CBR, payback period, annual CO₂ saving	Complete techno-economic assessment for Jordan

## Methodology

### Experimental site

All experiments were carried out at the Mechanical Engineering Department of Al-Zaytoonah University, Amman, Jordan (31.83°N, 35.89°E, elevation 820 m).

The site was selected for three reasons. First, the solar resource is substantial: the annual daily average irradiance on a south-facing vertical surface at this latitude is between 4 and 7 kWh/m², with more than 300 clear-sky days per year^26^. Second, the cool winter climate, with daytime ambient temperatures of 5–20 °C and cold nights, creates conditions that highlight the dynamic-insulation function of TSCs. Third, the prevailing wind during January-March averaged 1.0–1.9 m/s^27^, which is below the approximately 2 m/s level above which wind can significantly degrade unglazed collector performance^21^.

Experiments were conducted from January to March 2025, and only clear-sky days with V ∞ ≤ 2 m/s were accepted for the main dataset. This wind restriction was adopted as a control condition for the comparative objective of the study and is discussed further in Sect. 2.4.

### Module design and construction

A sealed test room (floor area 4 m²) was constructed against the south-facing exterior wall of the laboratory. All internal walls and the ceiling were insulated with extruded polystyrene (XPS) boards of known thickness and thermal conductivity; the south wall remained as the sole heat-exchange boundary. This configuration concentrates all heat exchange at the south face, eliminates lateral heat losses and allows a well-defined energy balance for each TSC module independently.

Two TSC modules were mounted side-by-side on the south wall. Both absorber plates were laser-machined from 2 mm galvanised steel sheet, 3.0 m high and 2.0 m wide (collector area A = 6.0 m² per module). The perforation pattern was a straight-line (square) arrangement of circular holes with a diameter of 1 mm and a centre-to-centre pitch of 15 mm, producing approximately 22,400 perforations and a nominal porosity of ϕ ≈ 0.3%. Both outer surfaces were coated with matt-black heat-resistant paint (solar absorptance α ≥ 0.95, long-wave emittance ε ≥ 0.93). An air plenum 110 mm deep separated each absorber plate from the back wall, in accordance with the recommended range of 100–150 mm for sufficient pressure recovery and flow uniformity^29^.

The UTSC module has no cover; its absorber face is directly exposed to outdoor ambient conditions. The GTSC module incorporates a single 4 mm tempered low-iron glass pane (solar transmittance τ = 0.91 at normal incidence; long-wave emittance ε_g_ = 0.84) mounted 30 mm in front of the absorber on anodised aluminium spacers, forming a sealed glass-to-absorber cavity.

**Fig. 1:**
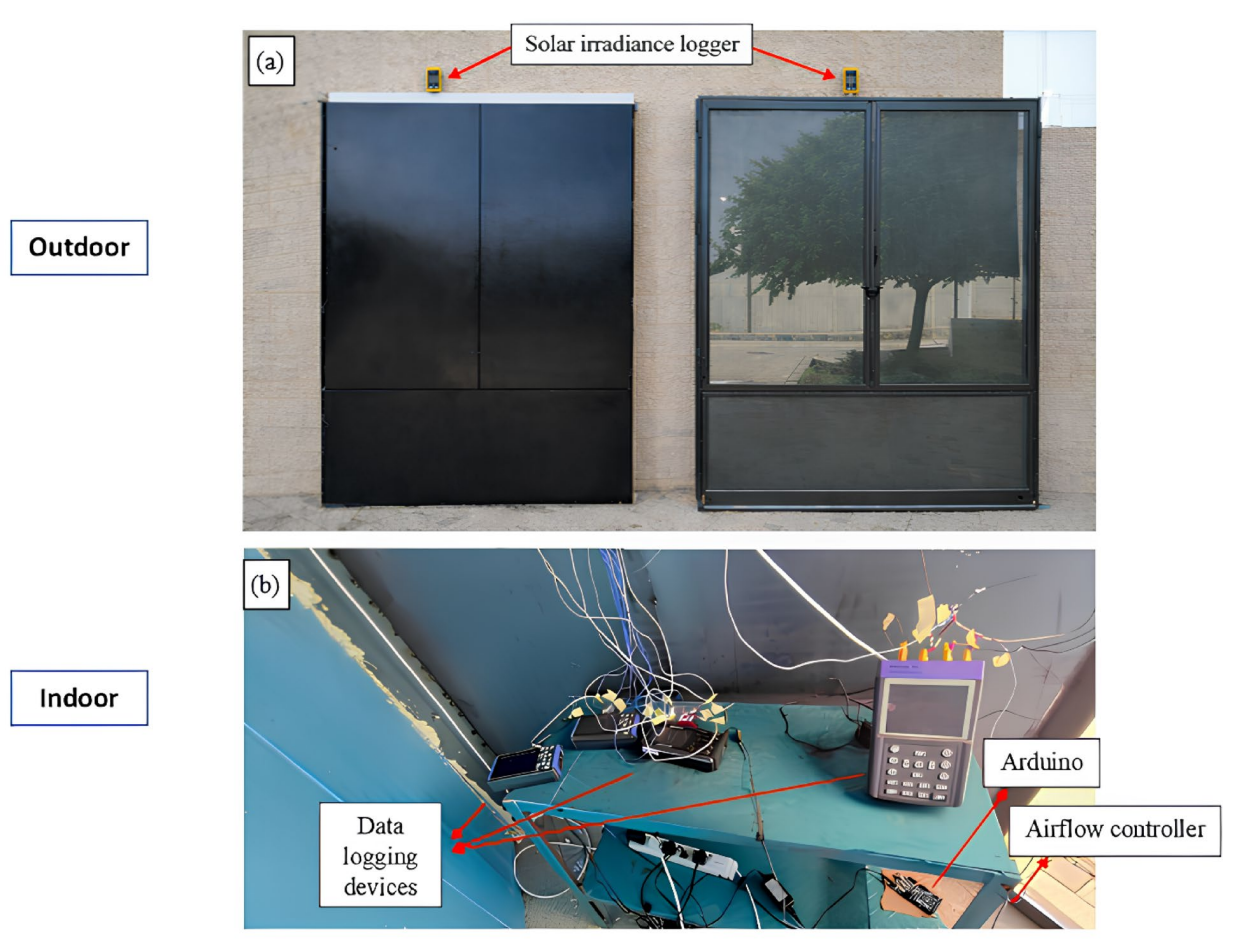
Experimental installation of the two modules at Al-Zaytoonah University, Amman. (**a**) Front view of the UTSC and GTSC mounted side-by-side on the south-facing façade; the GTSC used the same perforated absorber geometry as the UTSC, with a 4 mm low-iron glass cover installed 30 mm in front of the absorber. (**b**) Indoor view of the instrumentation, data-acquisition, and airflow-control system used during the experiments.

The 30 mm gap was adopted from the experimentally verified optimum by Mahmood^25^, which minimises internal convective loss without causing cavity overheating. Silicone gaskets sealed the glass perimeter to prevent ambient air infiltration into the cavity. For natural-convection tests, aluminium duct extension sections were attached above the module in steps to vary the effective cavity height over five values: 1.0, 1.5, 2.0, 2.5 and 3.0 m (reference height H_ref = 1.0 m). The overall outdoor/indoor arrangement is shown in Fig. 1, which presents the UTSC and GTSC mounted side-by-side on the same south-facing façade.

### Instrumentation

Temperature was measured using calibrated K-type thermocouples with a combined accuracy of ± 2.2 °C or ± 0.75% of reading, whichever is greater. Thirty sensors were embedded in each absorber backplate at 8 cm vertical intervals from the lower edge to the fan inlet. This arrangement provided a high-resolution temperature profile along the full plate height. The selected spacing represented a practical balance between measurement resolution and installation complexity, consistent with the approach described by Wang et al.^30^ for similar plate geometries.

**Fig. 2:**
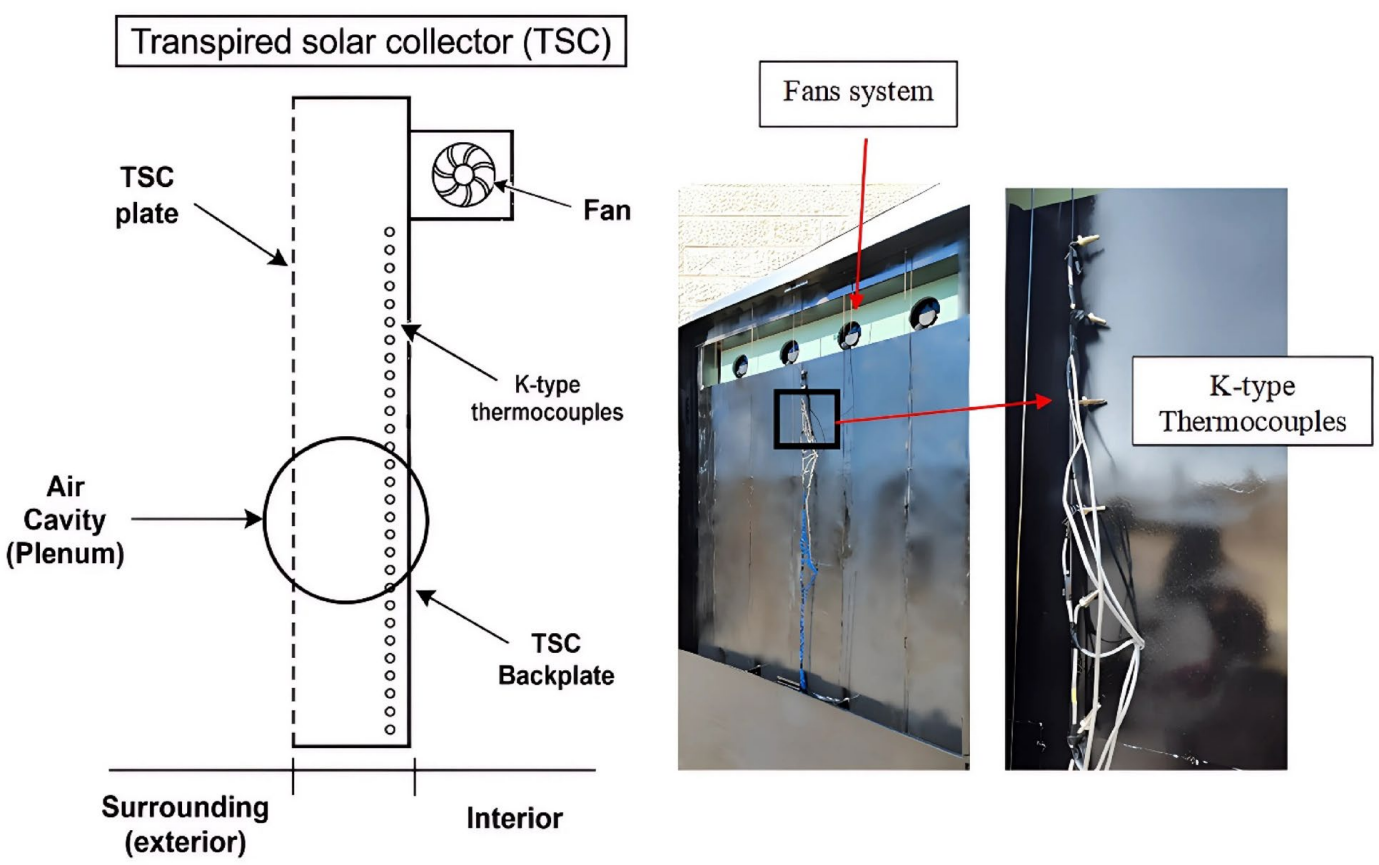
Cross-sectional view of the experimental TSC module and views of the plenum region in the installed module. Left: cross-sectional view showing the absorber plate, backplate, plenum, K-type thermocouple locations, and top-mounted fan. Right: views of the installed module showing the fan system and the arrangement of the installed K-type thermocouples used for temperature measurements.

Figure 2 presents the TSC configuration and the main experimental components, including the fan system, thermocouple layout, wall heat-flux measurement sections, and the indoor enclosure used as the interior boundary for the comparative tests. Three additional thermocouples were installed to monitor the absorber front-surface temperature, while one thermocouple measured the inlet air temperature at the base of the plenum. Indoor enclosure temperature was recorded at mid-height using three thermocouples. These measurements were used to monitor the enclosure thermal response and to support the assessment of interior boundary stability during the accepted steady-state periods.

All 37 channels were logged by four dedicated data loggers sampling synchronously at 5-minute intervals. For the GTSC, two additional surface-mounted K-type thermocouples were attached to the inner and outer glass surfaces to enable a complete glass-pane energy balance.

Air velocity within the plenum was measured using a Centre 332 hot-wire anemometer (velocity range 0–30 m/s, accuracy ± 3%; temperature range − 20 to 60 °C, accuracy ± 0.8 °C). The probe was introduced through ten measurement ports machined into the side plate of each module at regular height intervals, enabling direct velocity profiling within the cavity at multiple heights - the approach validated by Wang et al.^30^ for the same plate geometry.

Solar irradiance on the façade plane was monitored continuously by a Solar Survey 200R irradiance meter (range 0–1500 W/m²; resolution 1 W/m²; accuracy ± 2% of reading). The position of the solar irradiance meter relative to the two collectors is shown in Fig. 1. For natural-convection tests, the cavity outlet velocity was profiled on a 3 × 3 grid using the same hot-wire anemometer to obtain the area-averaged volumetric flow rate. A summary of all instruments appears in Table 2.

**Table 2:** Instrumentation specifications.

Measured quantity	Instrument	Range	Accuracy
Backplate temperature − 30 points [°C]	K-type thermocouple	−200 to + 1250 °C	± 2.2 °C or ± 0.75%
Absorber/glass surface temperature [°C]	K-type surface thermocouple	−200 to + 1250 °C	± 2.2 °C
Inlet air and indoor temperature [°C]	K-type thermocouple (4 loggers)	−200 to + 1250 °C	± 2.2 °C
Air velocity within plenum [m/s]	Centre 332 hot-wire anemometer	0–30 m/s	± 3%
NC outlet velocity [m/s]	Centre 332 (3 × 2 grid)	0–30 m/s	± 3%
Solar irradiance G [W/m²]	Solar Survey 200R irradiance meter	0–1500 W/m²	± 2%
Data recording	4 synchronised loggers	-	5-min interval

### Airflow system and test protocol

Forced airflow through each module was generated by four SUNON A2175HBT.TC.GN axial fans (120 mm diameter, 230 V AC, 26 W rated, maximum 344 m³/h each) managed by an Arduino-based variable-speed controller. The position of the fan outlets relative to the two collectors is shown in Fig. 2. The specific airflow rate, q_s, the area-normalised volumetric flow rate defined in Eq. (1), was set to four values: 18, 36, 54, 72, 90, 108, 126 and 144 m³/(h·m²), spanning the operationally recommended range for TSCs^29,31^.1

Experiments were conducted between 08:00 and 18:00 local solar time on clear-sky days selected by excluding any day with cloud cover, precipitation or V ∞ > 2 m/s (measured by a reference cup anemometer mounted at roof level). The restriction to clear-sky, low-wind, and quasi-steady periods was applied deliberately because the main aim of the study was to isolate the effect of glazing in a side-by-side comparison between the GTSC and the UTSC under matched boundary conditions. Since the unglazed configuration is more sensitive to wind-induced external convective losses, controlling the wind condition reduced a major source of environmental confounding and allowed the measured differences to be attributed more reliably to the collector configuration itself.

The absorber geometry used in both modules, namely 1 mm diameter perforations on a 15 mm square pitch with nominal porosity of approximately 0.3%, was selected as an optimised pattern for stable suction operation and wind-resistant behaviour^31,32^. In addition, the fan-assisted extraction system was used to promote uniform airflow over the full absorber surface. For the same absorber concept and operating framework, previous work by the authors confirmed compliance with the plate pressure-drop criterion commonly adopted to reduce wind-induced outflow and performance distortion^28,32^.

Before accepting a data point, a 20-minute steady-state period was required, during which the standard deviation of successive 5-minute averages had to remain below 1% of the mean for all thermocouple channels. Three representative conditions were repeated on independent days to assess repeatability; the resulting day-to-day scatter did not exceed 2% for thermal efficiency. For natural-convection experiments, the fans were turned off and a 60-minute stabilisation period was observed at each cavity height setting before recording.

To support repeatability, the geometric layout, sensor arrangement, auxiliary-equipment positions, and wall heat-flux measurement sections are summarised in Figs. 1 and 2. The side-wall ports used for plenum velocity measurements and the 3 × 2 outlet grid used in natural-convection tests are also indicated in the schematic representation.

### Performance metrics

Six performance metrics are defined below. Physical bounds are stated where they exist; these bounds follow directly from the definitions and require no additional assumptions.

#### Useful heat gain and thermal efficiency

The rate of useful heat delivered to the ventilation air stream, Q_u, is computed from the measured specific airflow rate, air density, specific heat and temperature rise across the collector:2

Where ρ = 1.20 kg/m³ at 20 °C and 900 hPa (representative of Amman at 820 m a.s.l.), and c_p = 1005 J kg⁻¹ K⁻¹. Thermal efficiency relates Q_u to the total incident solar energy on the collector face:3

Where G [W/m²] is the solar irradiance measured on the façade plane. A cosine correction for angle of incidence was applied using the solar position algorithm from the measured façade orientation and the site latitude.

#### Heat exchange effectiveness

The heat exchange effectiveness ε measures how closely the outlet air temperature approaches the absorber plate temperature:4

Where T_p [°C] is the spatial average of the 30 backplate thermocouple readings, representing the mean absorber temperature. By definition, ε ∈ (0, 1].

In the present study, ε is used as an instantaneous steady-state heat-transfer indicator for comparing collector behaviour under matched boundary conditions. It quantifies how closely the outlet air temperature approaches the mean absorber temperature during flow through the collector.

Under the present operating conditions, ε depends primarily on specific airflow rate and absorber geometry rather than on seasonal time variation. It is therefore not intended to represent a time-integrated daily or seasonal performance metric. Accordingly, ε describes the thermal coupling between the airflow and the absorber, whereas η describes the fraction of incident solar energy converted to useful heat^13,15,30^.

#### Exergy efficiency

The exergy efficiency ψ evaluates collector performance on a thermodynamic quality basis by accounting for the irreversibility of the solar-thermal conversion:5

Where T_a [K] and T_out [K] are absolute temperatures, and T_sun ≅ 4333 K is the apparent solar-source temperature adopted in the present simplified exergy calculation, accounting for atmospheric attenuation of solar radiation. All temperatures in Eq. (5) are absolute.

#### Wall heat-loss recapture index (UTSC)

The wall heat-loss recapture index quantifies the fractional reduction in conductive wall heat loss attributable to the dynamic-insulation action of the UTSC:6

Where q_cond, ref [W/m²] is the conductive heat flux through an adjacent unshielded reference wall section of identical construction, derived from thermocouple temperature differences across the known wall thickness using Fourier’s law, and q_cond, TSC is the flux through the wall section behind the UTSC. In the present sign convention, outward heat loss from the enclosure to the ambient environment is taken as positive. RI was evaluated only for accepted steady-state periods in which q_cond, ref > 0, meaning that the reference wall section was losing heat outward. Under this condition, RI is physically meaningful and strictly bounded in [0%, 100%]: RI = 0% means no reduction in wall heat loss; RI = 100% means complete elimination.

The measurement arrangement used for the UTSC wall heat-loss recapture analysis is shown in Fig. 2. The conductive heat fluxes were determined from Fourier’s law as follows:7a7b

Where k_w is the wall thermal conductivity, δ_w is the wall thickness, q_cond, ref is the conductive heat flux through the adjacent unshielded reference wall section, and q_cond, TSC is the conductive heat flux through the wall section located behind the UTSC. Both fluxes were checked using the measured wall-temperature differences, and only outward reference-wall heat-loss windows were retained for the RI analysis.

#### Ventilation load reduction (GTSC)

The ventilation load reduction quantifies the fraction of supplementary heating demand eliminated by the GTSC:8

Where P_heat, ref = q_s ρ A c_p max(T_sp − T_amb, 0) is the supplementary heating power required without the collector, and P_heat, GTSC = q_s ρ A c_p max(T_sp − T_out, 0) is the residual supplementary heating required with the GTSC. Here, T_sp [°C] is a reference supply-air heating set-point, fixed at 22 °C in the present study, T_amb [°C] is the measured outdoor ambient air temperature, and T_out [°C] is the collector outlet air temperature. VLR is therefore a collector-boundary ventilation-air preheating metric derived from the outlet-air condition. It should not be interpreted as proof that the whole test enclosure reached or maintained 22 °C.

#### Cost-benefit ratio

The cost-benefit ratio provides a simple but consistent economic performance index:9

A higher CBR reflects a greater annual monetary return per unit of capital invested. The simple payback period in years equals 1/CBR.

### Uncertainty analysis

Combined measurement uncertainties were propagated from individual instrument specifications using the root-sum-of-squares (RSS) method. In the original submission, uncertainty values were reported for one representative operating point only. To provide a more complete description of measurement confidence, the uncertainty analysis was expanded to selected low, medium, and high operating conditions spanning the tested domain. Table 3 summarises the representative measured values together with their propagated combined uncertainties.

As expected, relative uncertainty increases when ΔT becomes small, because the fixed thermocouple uncertainty represents a larger fraction of the measured temperature rise. Across the selected operating cases, uncertainty decreases as solar irradiance increases and tends to be larger at higher specific airflow rates, where the resulting temperature rise is smaller. The low-irradiance case in Table 4 provides the requested low-ΔT example and shows the largest propagated uncertainty. By contrast, the mid- and high-irradiance cases show substantially lower relative uncertainty because the measured thermal signal is stronger.

**Table 3:** Representative measured values and propagated combined uncertainties at selected operating conditions spanning the tested domain.

Operating condition	G [W/m²]	q_s [m³/(h·m²)]	η [%]	ε [-]	ψ [%]	RI (UTSC) [%]	VLR (GTSC) [%]	ΔT [°C]
Low irradiance Low-flow case	200–400	18–54	± 6.0	± 0.045	± 0.8	± 6	± 5	± 2.2
Reference condition	400–800	54–108	± 4.8	± 0.035	± 0.5	± 4	± 4	± 0.8
High irradiance High-flow case	800–1000	108–144	± 2.0	± 0.033	± 0.4	± 4	± 3	± 0.8

Note: Reported values are propagated combined uncertainties obtained by the root-sum-of-squares (RSS) method using the instrument specifications listed in Table 2. The selected cases represent low-, mid-, and high-end operating conditions across the tested domain. The reference condition is the same operating point used for the figure error bars.

### Analytical model

A one-dimensional steady-state analytical model was developed to extend predictions beyond the directly tested parameter space and to serve as a physically consistent interpolation tool. The model solves coupled energy balances on five control volumes - the outdoor ambient, the glass pane (GTSC only), the absorber plate, the plenum air and the building wall - incorporating convective, conductive and long-wave radiative heat-transfer mechanisms at each interface. For the GTSC, long-wave radiative exchange between the absorber and the glass was treated as a two-surface enclosure.

Internal convection at the perforated absorber is described by the Kutscher-Van Decker correlation^13,15^:10

Where Nu = h_c d/k_air is the Nusselt number based on hole diameter d = 1 mm, Re = ρ v_approach d/µ is the hole Reynolds number, ϕ = 0.3% is porosity and *p* = 15 mm is pitch. This correlation was validated experimentally by both Kutscher^14^ and Van Decker et al.^15^ for hole diameters in the range 0.5–2 mm and porosities up to 2%, well within the geometry of the present absorbers.

For natural-convection operation, the buoyancy pressure driving the flow through the cavity was estimated from the stack-effect model:11

Where H [m] is cavity height and T_c [K] is the mean cavity air temperature. No pressure instruments were used in the experiment; ΔP in Eq. (11) is a model output used solely to interpret the measured velocity data. The model was solved iteratively with a convergence criterion of 10^− 6^ on all energy-balance residuals.

Validation against measurements yielded agreement within ± 10% for thermal efficiency across all tested conditions, which is consistent with the accuracy reported for the same correlation by Van Decker et al.^15^ under similar operating ranges. The validated analytical model was subsequently used as the seasonal integration tool for estimating annual useful heat delivery and the associated economic indicators reported later in the manuscript.

## Results

All figures include error bars derived from the RSS uncertainty analysis described in Sect. 2.6. Each data point represents the 20-minute steady-state average of 5-minute interval readings. The two modules were always tested simultaneously on the same day, so all UTSC-GTSC comparisons are made under identical ambient conditions.

### Heat exchange effectiveness

**Fig. 3:**
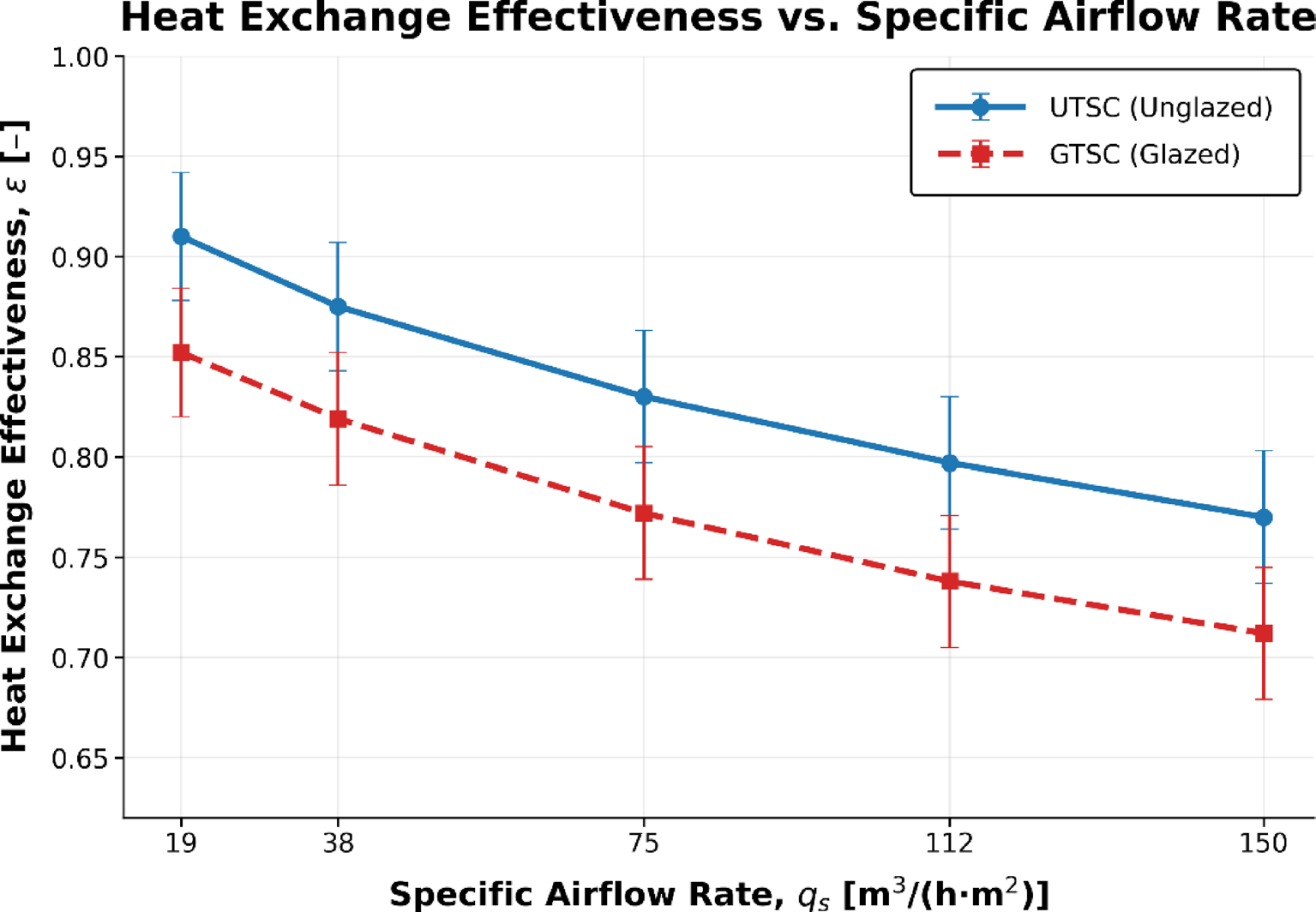
Heat exchange effectiveness ε as a function of specific airflow rate q_s for the UTSC and GTSC. In both systems, ε decreases as q_s increases because the air-plate contact time becomes shorter. The UTSC remains slightly higher than the GTSC across the full range.

Because ε is used here as an instantaneous indicator of airflow-absorber thermal coupling, it is reported as a function of specific airflow rate rather than as a seasonal performance quantity. Figure 3 shows the heat exchange effectiveness ε as a function of specific airflow rate for both collectors. At q_s = 18 m³/(h·m²), ε = 0.91 ± 0.033 for the UTSC and 0.85 ± 0.033 for the GTSC. Both values decline consistently as q_s increases, reaching 0.77 (UTSC) and 0.71 (GTSC) at q_s = 144 m³/(h·m²). The heat exchange effectiveness values were calculated from accepted steady-state records using simultaneous measurements of T_out_, T_in_, and the mean absorber temperature $\:{\stackrel{-}{\mathrm{T}}}_{\mathrm{p}}$, according to Eq. (4).

This inverse relationship with flow rate reflects the reduction in air-plate contact time at higher velocities, which limits the temperature rise per unit mass of air. The 0.06 gap between the two systems across the entire flow range is attributable to the additional thermal resistance introduced by the 30 mm glass-to-absorber cavity of the GTSC: the cavity air is more stagnant and thermally decoupled from the main plenum airstream compared with the directly exposed UTSC absorber. Notably, both systems maintain ε above 0.70 across the full tested range, confirming that the 1 mm hole, 15 mm pitch geometry provides adequate heat transfer at all practical flow rates.

### Thermal efficiency

#### Dependence on solar irradiance

**Fig. 4:**
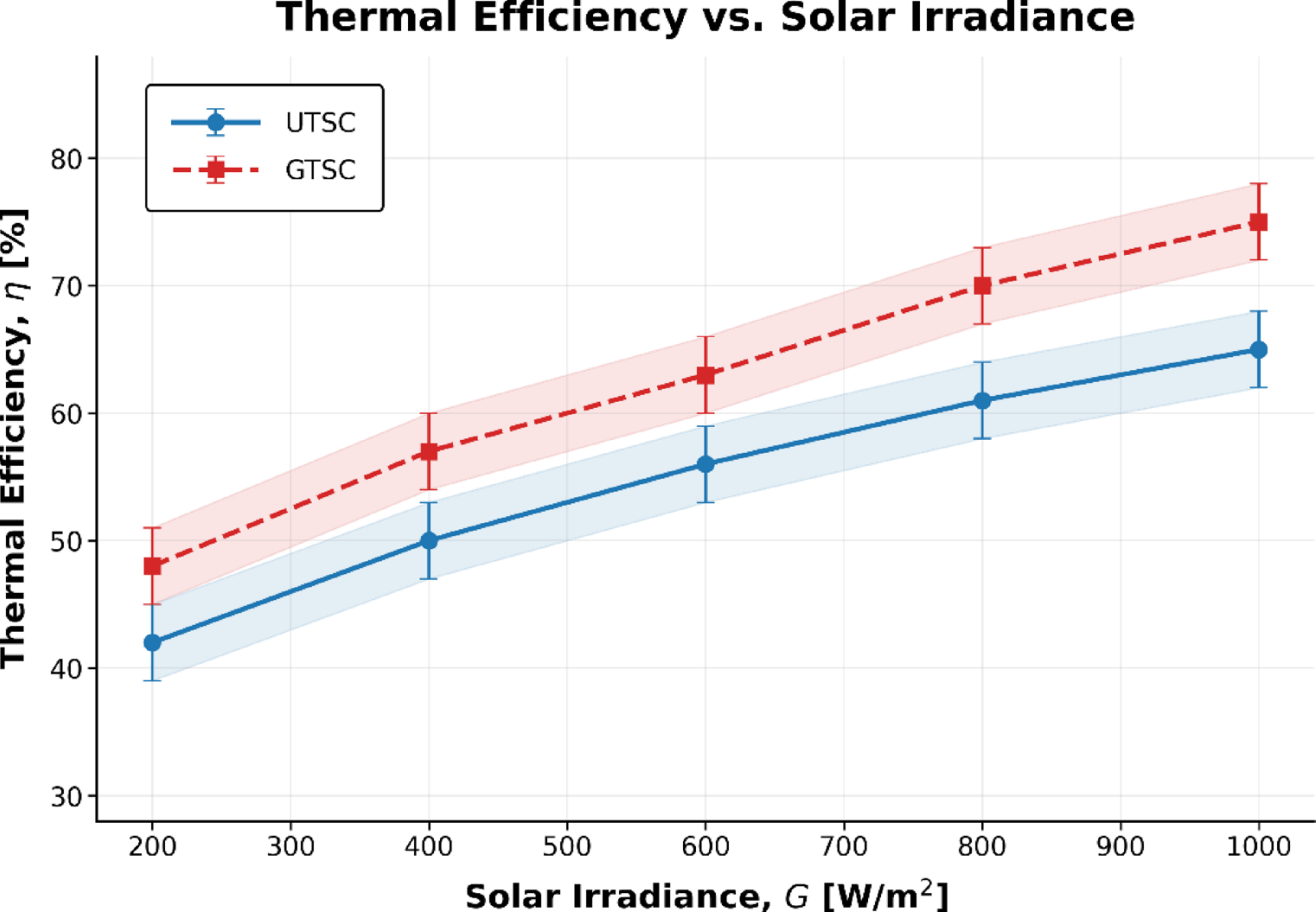
Thermal efficiency η as a function of solar irradiance G for both collector types at q_s = 90 m³/(h·m²). Thermal efficiency increases with irradiance for both systems, while the GTSC maintains a consistent advantage due to reduced convective and radiative heat losses.

**Fig. 5:**
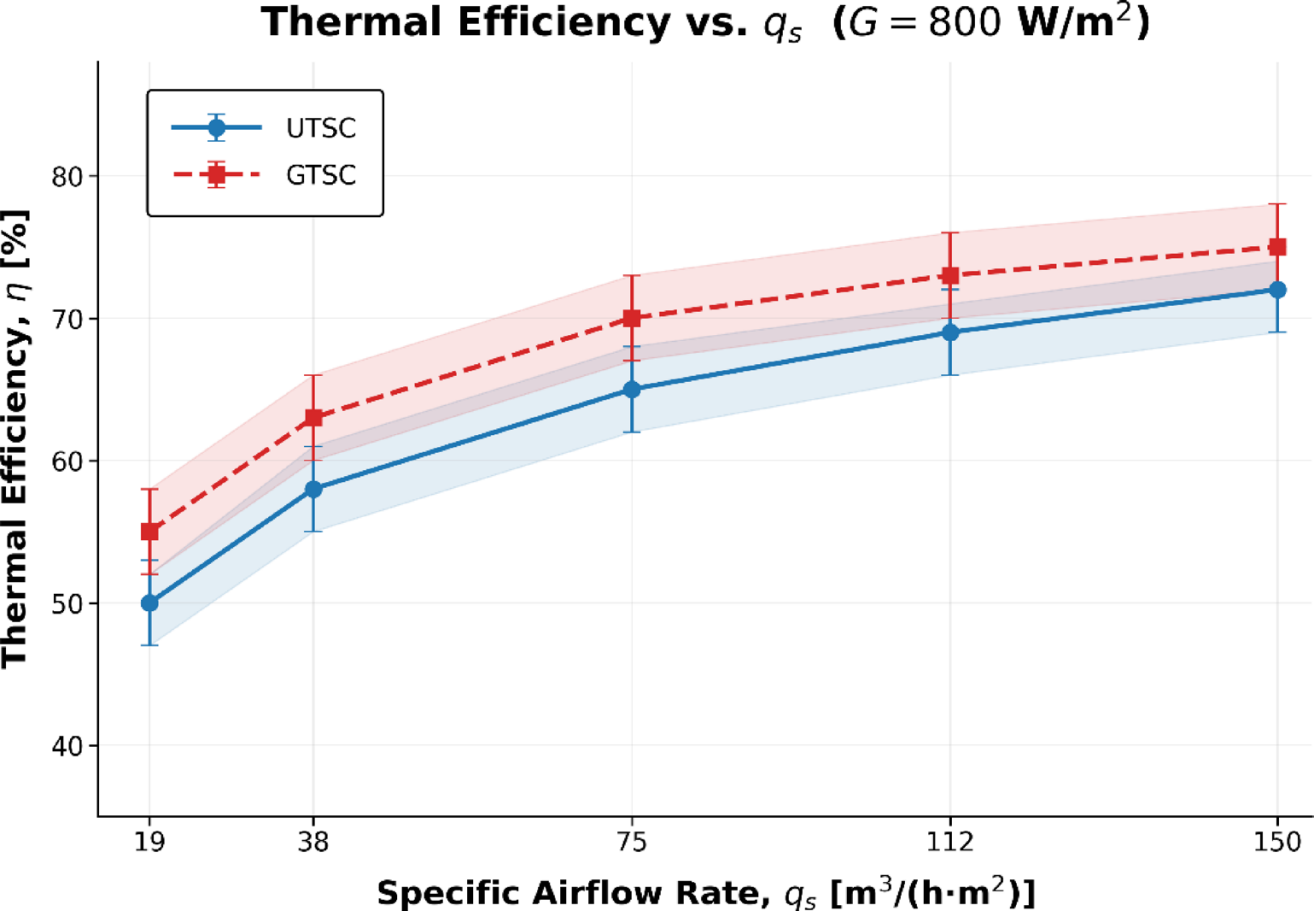
Thermal efficiency η as a function of specific airflow rate q_s at G = 800 W/m². Both collectors show a rapid increase in η at low q_s, followed by a plateau near q_s = 90 m³/(h·m²). The GTSC remains more efficient across the full operating range.

**Fig. 6:**
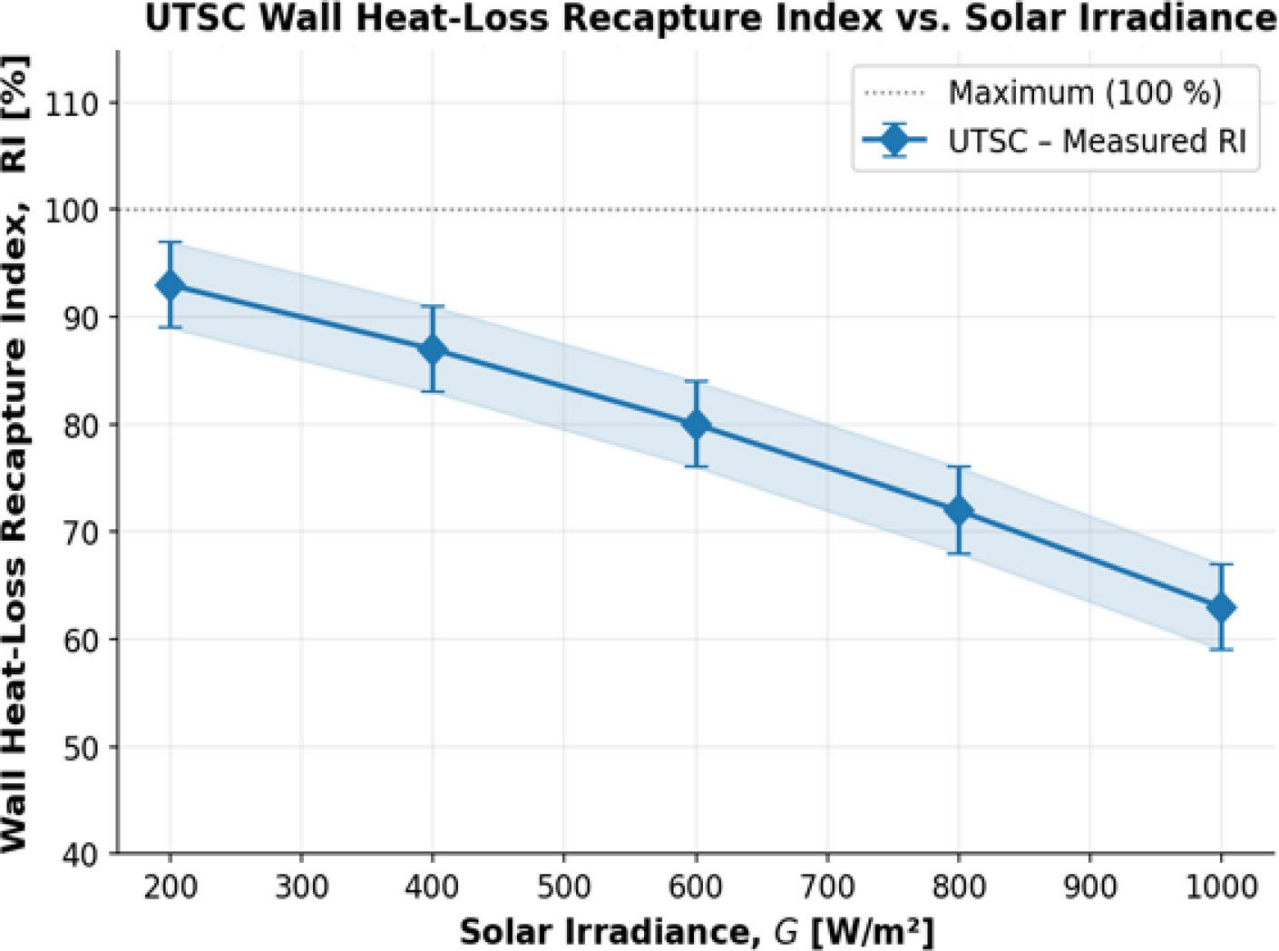
UTSC wall heat-loss recapture index RI as a function of solar irradiance G at q_s = 90 m³/(h·m²). RI decreases with irradiance, showing that dynamic-insulation behaviour is strongest under lower-irradiance conditions.

**Fig. 7:**
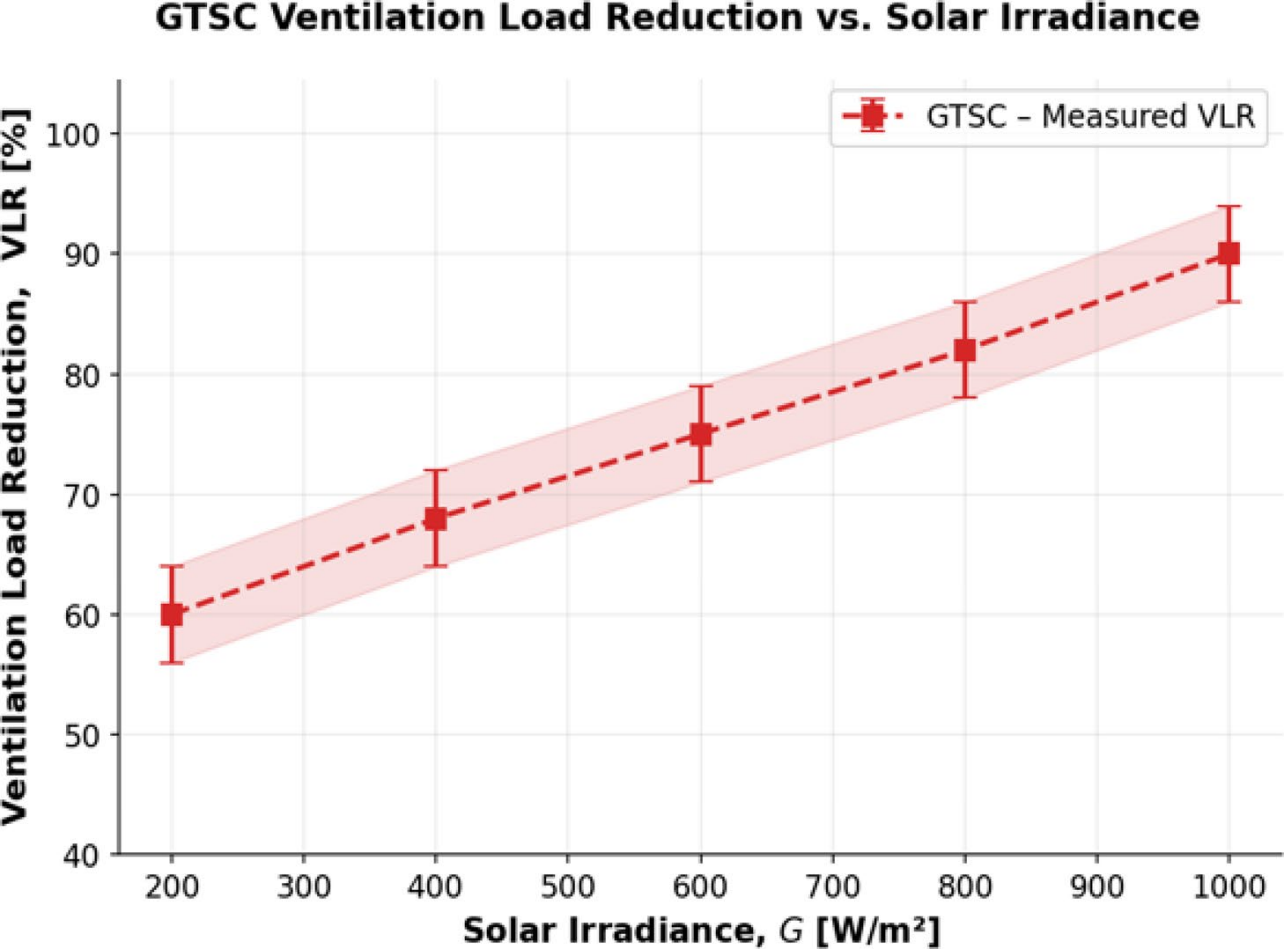
GTSC ventilation load reduction VLR as a function of solar irradiance G at q_s = 90 m³/(h·m²). VLR increases with irradiance because higher outlet air temperature reduces the calculated supplementary heating required to raise the delivered ventilation air to the reference supply-air set-point.

**Fig. 8:**
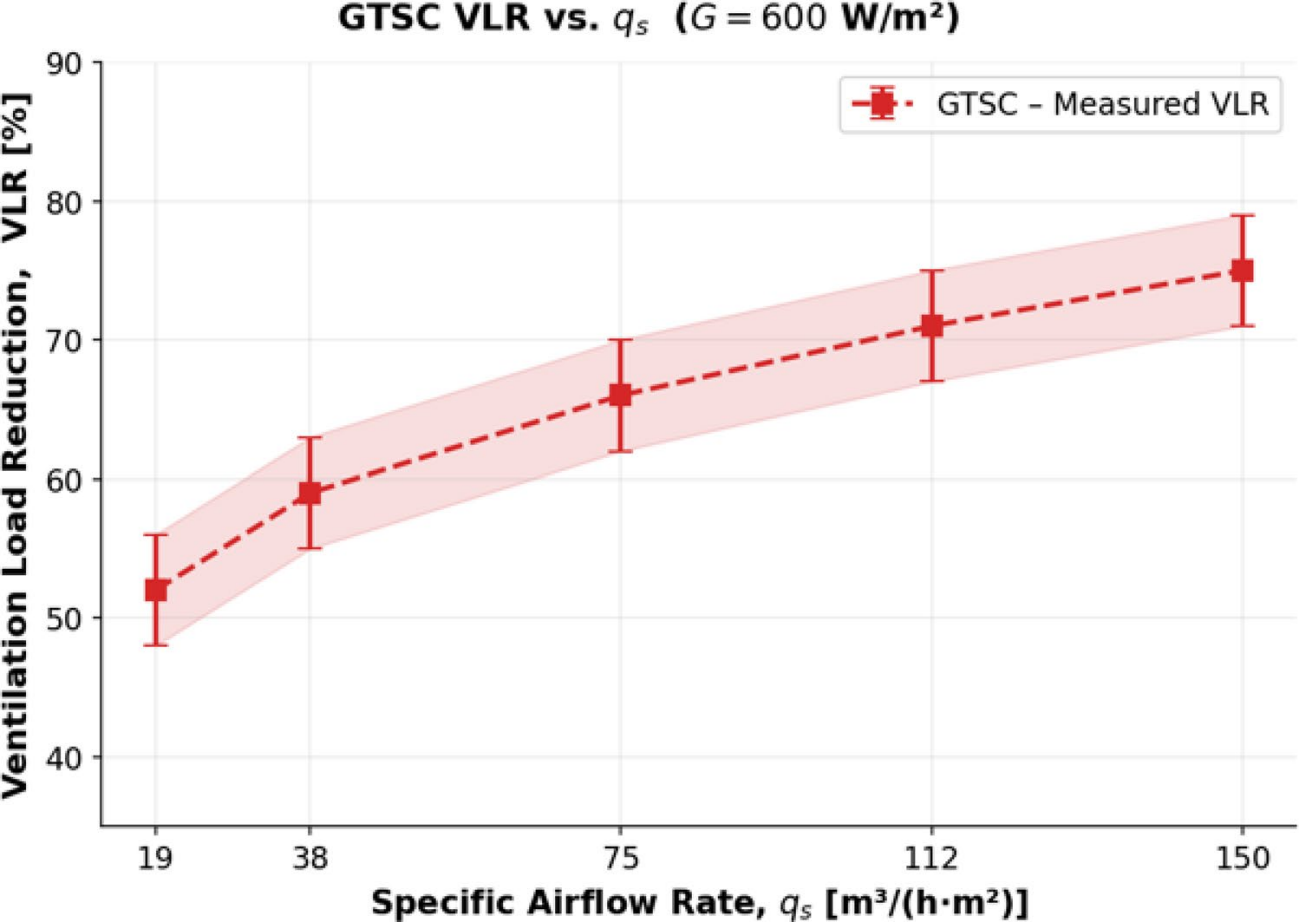
GTSC ventilation load reduction VLR as a function of specific airflow rate q_s (G = 600 W/m²).

**Fig. 9:**
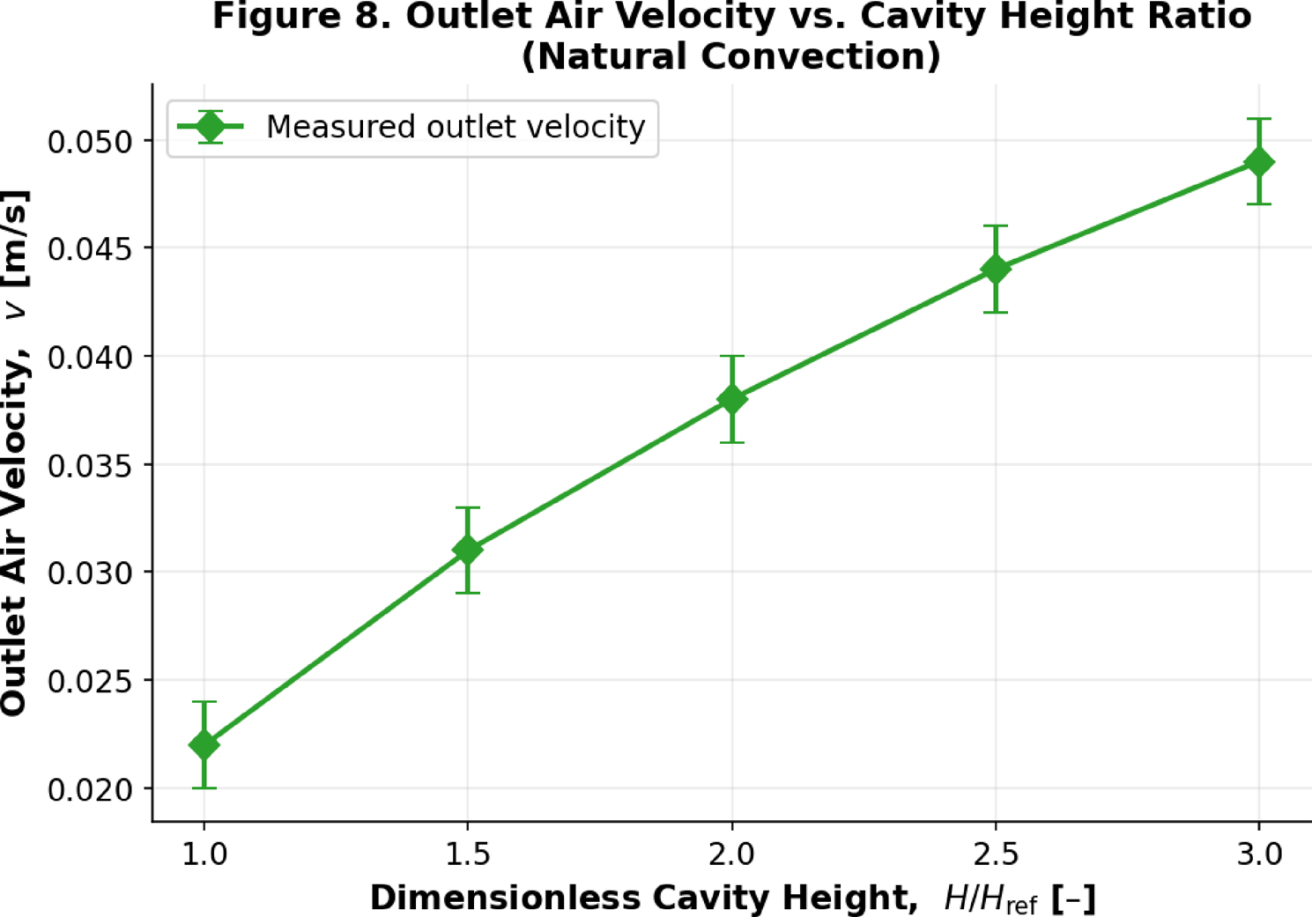
GTSC natural-convection outlet air velocity as a function of H/H_ref at G ≈ 600 W/m². Outlet velocity increases with cavity height because the buoyancy pressure difference becomes larger as the warm-air column grows.

**Fig. 10:**
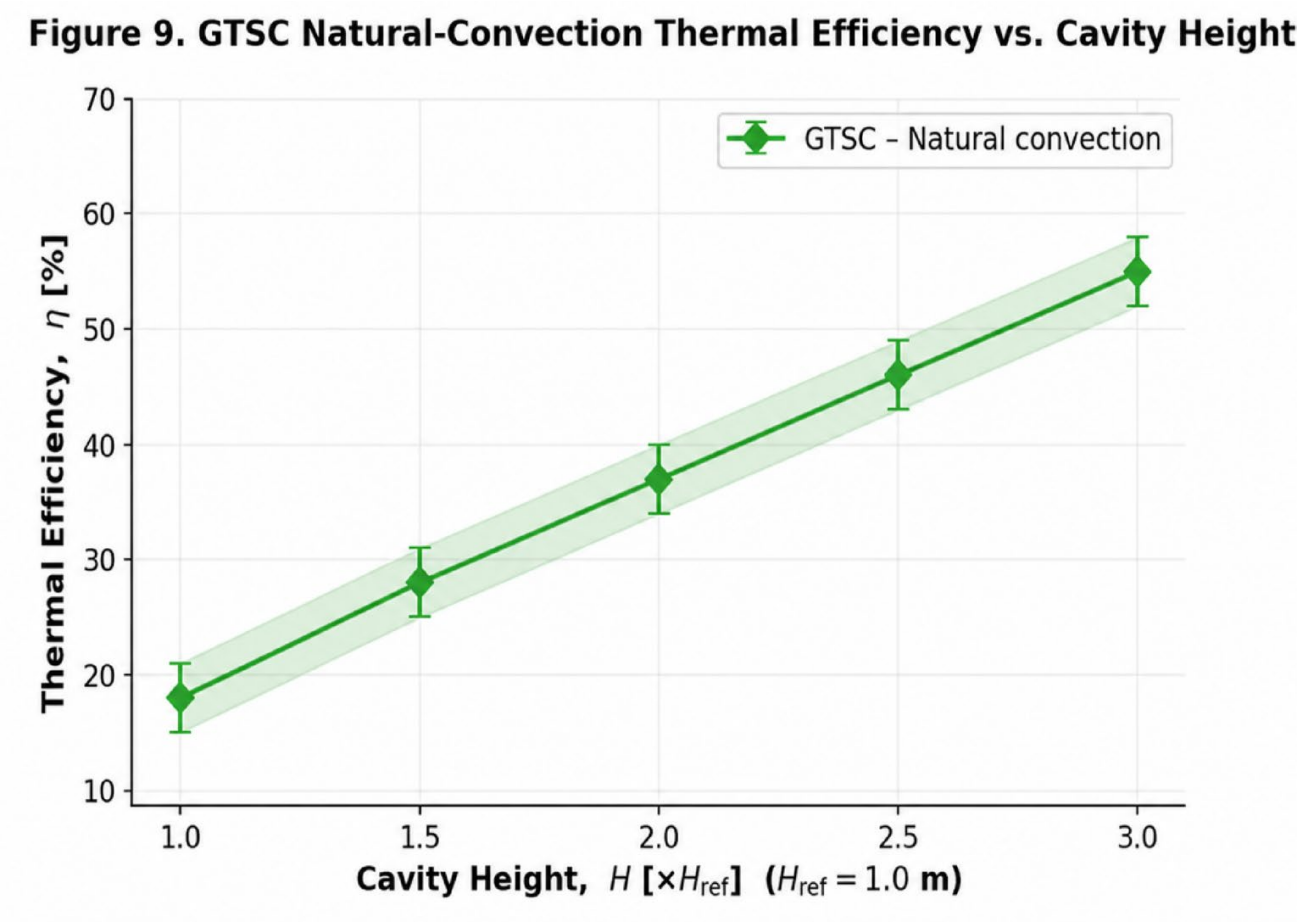
GTSC thermal efficiency under natural-convection operation as a function of H/H_ref at G ≈ 600 W/m². Thermal efficiency increases strongly with cavity height, demonstrating the growing benefit of buoyancy-driven airflow in passive operation.

**Fig. 11:**
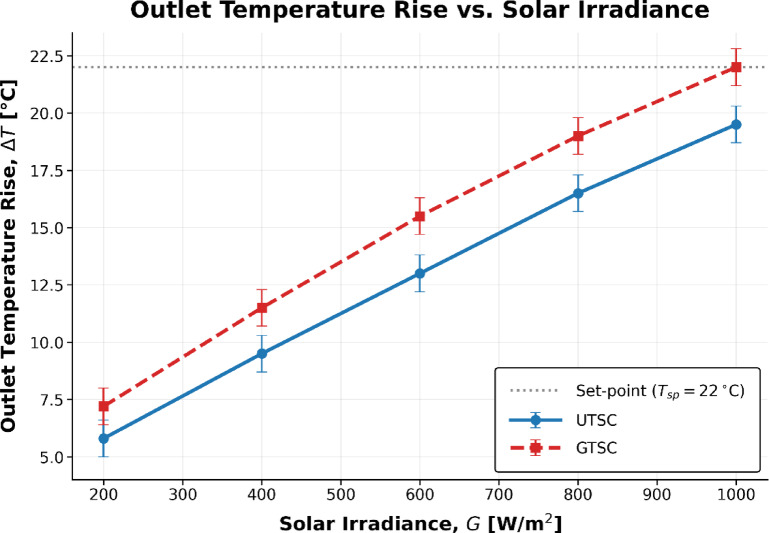
Outlet temperature rise ΔT as a function of solar irradiance G at q_s = 90 m³/(h·m²) for the UTSC and GTSC. The GTSC delivers a consistently higher outlet temperature rise than the UTSC across the full irradiance range. The dotted line marks the 22 °C reference outlet-temperature rise used in the VLR calculation.

**Fig. 12:**
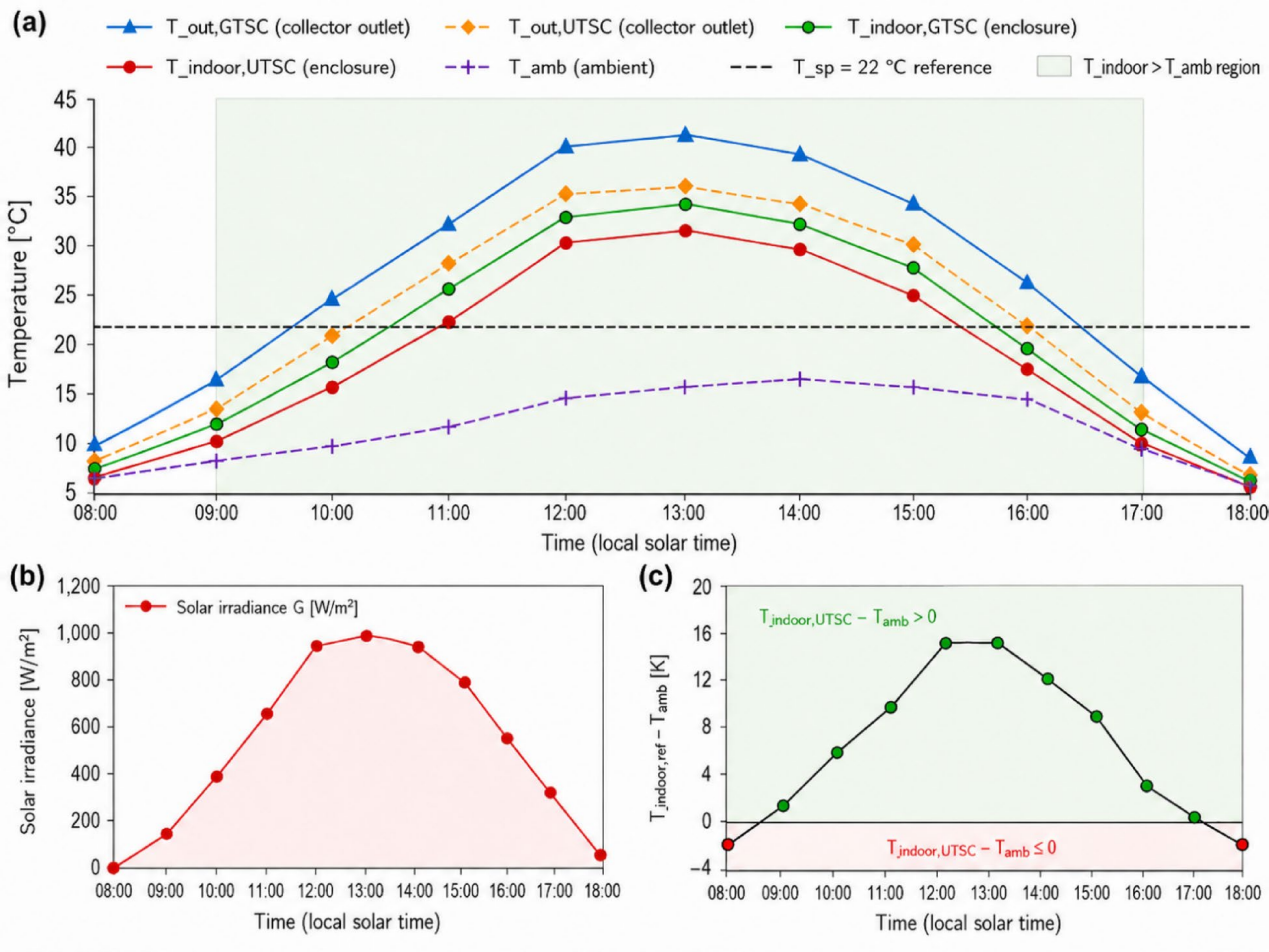
Representative clear-sky test-day enclosure response and solar input. (**a**) Indoor enclosure, collector outlet, and ambient temperatures; the dashed $\:{22}^{\circ\:}\mathrm{C}$ line denotes the reference supply-air set-point used in the VLR calculation. (**b**) Façade solar irradiance over the same period. (**c**) $\:{(\mathrm{T}}_{\mathrm{indoor}\mathrm{,\:UTSC\:}}-{\mathrm{T}}_{\mathrm{amb\:}})$ showing the period consistent with outward reference-wall heat loss during RI evaluation. Performance indices were evaluated from accepted 20 -min steady-state records.

**Fig. 13:**
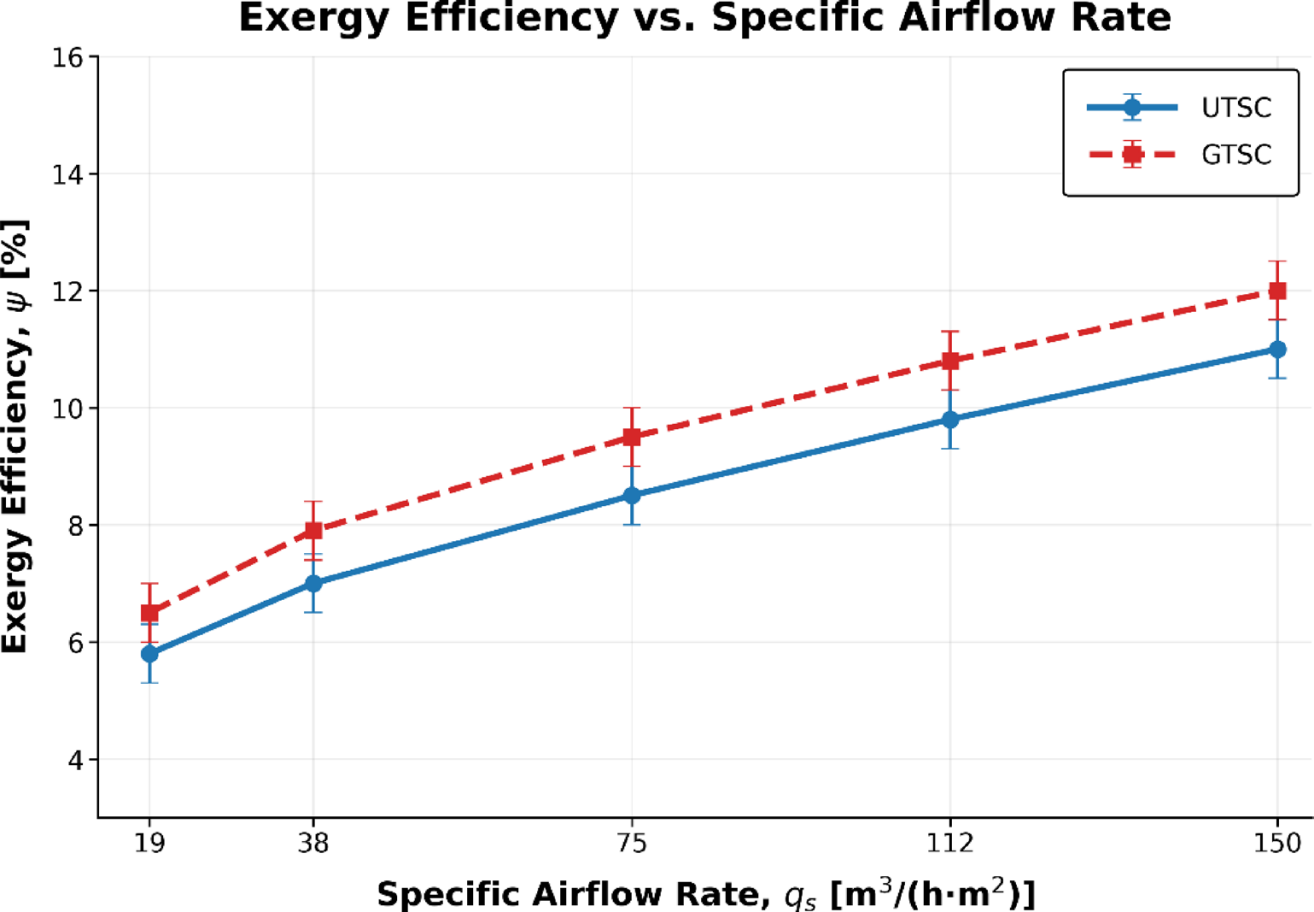
Exergy efficiency ψ as a function of specific airflow rate q_s.

**Fig. 14:**
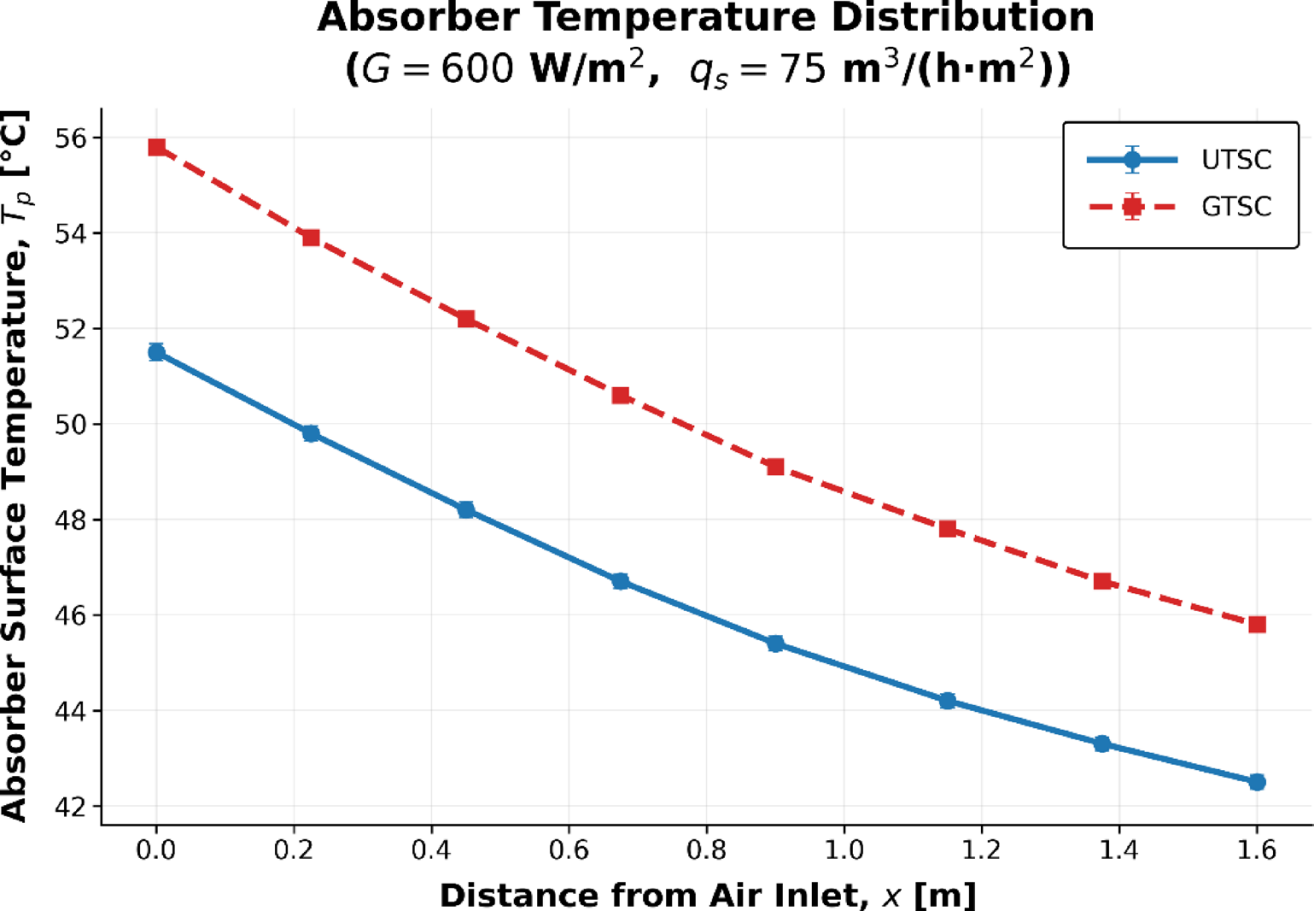
Absorber backplate temperature T_p along the plate height at G = 600 W/m², q_s = 90 m³/(h·m²).

**Fig. 15:**
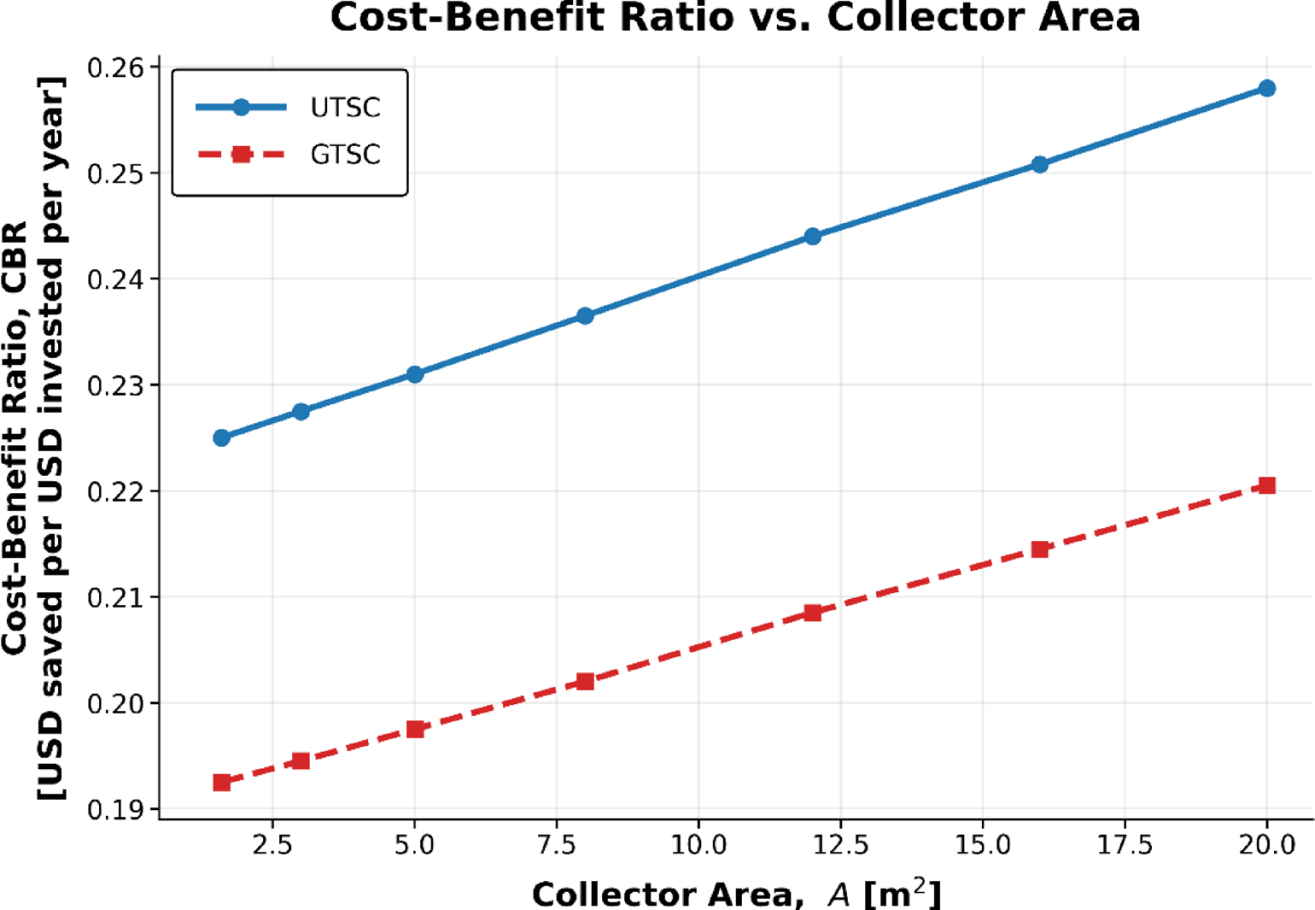
Cost-benefit ratio CBR as a function of total installed collector area for both systems. Higher CBR indicates a greater annual energy saving per dollar of capital invested.

Figures 4, 5, 6, 7, 8, 9, 10, 11, 12, 13, 14 shows η as a function of G at q_s = 90 m³/(h·m²), which is approximately the flow rate that maximises the combined exergy and thermal efficiency of both systems (see Sect. 3.6 and Fig. 15). For the UTSC, η rises from 42 ± 3% at G = 200 W/m² to 65 ± 3% at G = 1000 W/m²; for the GTSC, from 48 ± 3% to 75 ± 3% over the same range. The increasing trend with irradiance arises because higher G raises the absorber temperature relative to ambient, increasing the driving force for heat transfer to the suction air and reducing the fractional share of losses in the total energy budget. The consistent 6–9% point GTSC advantage reflects the suppression of external convective heat loss from the absorber by the glass cover and the reduction of long-wave radiative emission to the sky. At all irradiance levels, these loss-reduction gains outweigh the 9% optical penalty of the glass. The measured GTSC efficiency range agrees well with the 48–75% reported by Mahmood^25^ under comparable semi-arid conditions, and the relative improvement over the UTSC is consistent with the 20% gain found by Gao et al.^23^ for their glazed corrugated-plate design.

#### Dependence on specific airflow rate

Figure 5 shows η versus q_s at G = 800 W/m². Both curves rise steeply from q_s = 18 m³/(h·m²) and flatten into a plateau near 90 m³/(h·m²), above which additional flow rate yields diminishing efficiency gains: the absorber cools more rapidly but the lower temperature rise per unit mass offsets the benefit. The GTSC maintains a 3–5% point advantage throughout. The plateau region at q_s = 72–90 m³/(h·m²) represents the recommended operating zone: η > 65% (GTSC) with a useful outlet temperature rise exceeding 15 °C, achievable without over-sizing the fan system and incurring excessive electrical consumption.

The present study was designed as a controlled outdoor comparison, with the UTSC and GTSC tested simultaneously under matched boundary conditions. The reliability of the observed thermal-efficiency difference was therefore evaluated using repeated representative cases, propagated uncertainty analysis, and the consistent separation between the GTSC and UTSC results across the tested range. Formal full-factorial hypothesis testing at every operating point was not part of the experimental design.

### Dynamic insulation performance

#### Wall heat-loss recapture index (UTSC)

Figure 6 presents the UTSC recapture index RI as a function of solar irradiance at q_s = 90 m³/(h·m²). At G = 200 W/m², RI = 93 ± 4%. The suction flow at this condition maintains the outer wall surface close to ambient temperature, creating a large temperature difference between the warm interior wall face and the cool backplate face, which drives a strong heat recapture flux. As G increases, the absorber heats progressively above ambient, gradually reducing the temperature gradient and the associated driving force; RI falls monotonically to 63 ± 4% at G = 1000 W/m². This decreasing trend with irradiance is the physically correct behaviour for a dynamic insulator: wall heat-loss recapture is strongest when solar gain is modest and building heat losses are greatest, which corresponds to the early morning and cold-day conditions that are most critical for winter heating energy savings. All RI values lie within the physical bound [0%, 100%] consistent with Eq. (6).

#### Ventilation load reduction (GTSC)

Figure 7 shows the GTSC ventilation load reduction VLR as a function of solar irradiance. VLR rises from 60 ± 4% at G = 200 W/m² to 90 ± 4% at G = 1000 W/m². Because VLR is evaluated from the outlet-air heating requirement defined by Eq. (9), the monotonic increase with irradiance reflects the rise in T_out relative to the fixed 22 °C reference supply-air set-point. At G ≈ 980 W/m², the GTSC outlet-temperature rise approaches 22 °C, so the auxiliary heating required to lift the delivered ventilation air to the 22 °C reference becomes very small. This interpretation applies to the collector outlet stream and should not be read as a whole-enclosure comfort result.

Figure 8 shows VLR as a function of specific airflow rate at G = 600 W/m². VLR increases from 52% at q_s = 18 m³/(h·m²) to 75% at q_s = 144 m³/(h·m²). Higher volumetric flow delivers more pre-warmed air per unit time, reducing the calculated supplementary heating required for the ventilation air stream under the adopted VLR definition. Both the irradiance and flow-rate trends are monotonically increasing and consistent with one another, confirming the internal coherence of the measurements and the metric.

### Natural-convection operation

Figure 9 shows the area-averaged outlet air velocity at the cavity top as a function of dimensionless height H/H_ref (H_ref = 1.0 m) for the GTSC operating without fans. The velocity increases from 0.022 m/s at H = 1.0 m to 0.049 m/s at H = 3.0 m. This increase is explained by the growth of buoyancy pressure with cavity height described by Eq. (11): a taller column of warm air generates a larger pressure differential between the cavity base and the outlet, accelerating the flow. The model prediction (not shown for clarity) agrees with the measurements within 8% at all heights.

Figure 10 presents the natural-convection thermal efficiency. Starting at 18 ± 3% when H = 1.0 m, η reaches 55 ± 3% at H = 3.0 m. The 3.0 m efficiency is comparable to the forced-convection performance at the lowest tested flow rate (Fig. 3 at q_s = 54 m³/(h·m²)), demonstrating that a tall-enough glazed cavity can fully substitute fan-driven operation for single-storey residential façades. This result extends the passive TSC performance data reported for updraft tower geometries by Eryener et al.^33^ to compact, wall-integrated modules.

### Outlet temperature rise

Figure 11 shows ΔT = T_out − T_in as a function of solar irradiance at q_s = 90 m³/(h·m²). The GTSC delivers outlet temperatures 1.2–2.5 °C higher than the UTSC at all irradiance levels, consistent with its higher thermal efficiency. At G = 1000 W/m², the GTSC reaches ΔT = 22.0 ± 0.8 °C while the UTSC achieves 19.5 ± 0.8 °C. The dotted horizontal line marks the 22 °C reference outlet-temperature rise associated with near-complete elimination of supplementary heating in the VLR calculation. It is a collector-outlet reference, not a statement of whole-room temperature. The near-linear ΔT-G relationship across the entire range confirms that heat exchange is consistent and well-controlled with no evidence of thermal stratification or flow instability at any tested condition.

### Indoor enclosure temperature response

Indoor Indoor air temperature within the test enclosure was monitored throughout the campaign using three thermocouples placed at mid-height. Figure 12 presents a representative clear-sky test-day profile showing the temporal evolution of indoor enclosure temperature, collector outlet temperature, ambient temperature, façade solar irradiance, and the indoor-ambient temperature difference relevant to RI interpretation.

Figure 12 documents the transient response of the test enclosure and the interior boundary condition during the representative day. The enclosure air follows the collector outlet temperature with a clear thermal lag, which is expected in a forced-flow test arrangement. The delivered heat is distributed among enclosure air heating, transient heat storage in the enclosure surfaces and wall materials, heat exchange through the enclosure boundaries, and the ventilation flow path. Therefore, the enclosure air temperature cannot be interpreted using only the heat capacity of the air volume.

Figure 12 (c) shows that $\:{(T}_{\mathrm{indoor,\:UTSC\:}}-{T}_{\mathrm{amb\:}})$ remained positive during the principal RI evaluation period. This confirms that the UTSC reference-wall section was under outward heat-loss conditions during the relevant steady-state records. The RI values reported in Fig. 6 were calculated from measured conductive wall heat fluxes through the reference wall section and the wall section behind the UTSC, rather than from the bulk indoor and ambient air temperatures alone.

For the GTSC case, Fig. 12 (a) shows a stronger enclosure response during the high-irradiance period, consistent with the higher outlet temperature of the glazed configuration. The main comparison between the UTSC and GTSC, however, is based on collector-boundary and wall-flux quantities, including outlet temperature rise, heat exchange effectiveness, thermal efficiency, wall heat-flux reduction, and calculated ventilation-air preheating reduction.

The quantitative performance indices were evaluated from the accepted steady-state records for each operating condition, using simultaneous measurements of airflow rate, inlet and outlet air temperatures, absorber temperature, solar irradiance, and wall heat flux. Figure 12 therefore provides the time-dependent enclosure-response context for the representative clear-sky day, while the UTSC-GTSC performance comparison is based on the steady-state measurements reported in the corresponding results figures.

### Exergy efficiency

Figure 13 shows exergy efficiency ψ versus specific airflow rate. Both systems display a monotonically increasing trend: at higher flow rates, more total heat is extracted per unit time, raising both the absolute temperature rise and the exergy content of the delivered air stream per unit of incident solar radiation. Peak values at q_s = 108 m³/(h·m²) are ψ = 12.0 ± 0.5% (GTSC) and 11.0 ± 0.5% (UTSC). The low absolute values - well below 15% - reflect the thermodynamic limitation common to all solar air heaters: solar radiation enters as high-temperature exergy (≅ 4333 K effective temperature) but is delivered as warm air only slightly above ambient, generating unavoidable entropy in the conversion process^34–36^. The GTSC advantage of 1–1.5% points arises because suppressed losses mean the air stream is delivered at a marginally higher temperature, carrying slightly more exergy per unit mass. The interaction between G and q_s on ψ is explored further through the contour map in Fig. 15.

### Absorber temperature distribution

Figure 14 shows the absorber backplate temperature profile along the plate height for both modules at G = 600 W/m² and q_s = 90 m³/(h·m²). Air enters the plenum at the bottom (x = 0) and is extracted at the top (x = 3.0 m) by the fans. In both modules, T_p decreases nearly linearly from inlet to outlet as suction air draws heat from the plate progressively along the flow path. The GTSC absorber maintains temperatures 4–6 °C higher than the UTSC throughout the height; this elevated operating temperature is the direct consequence of the glass cover retaining heat within the cavity, and it explains the higher thermal efficiency seen in Figs. 3 and 4. The near-linear temperature gradient is consistent with the uniform solar flux assumption underlying the one-dimensional model and validates the energy balance used to derive Eqs. (2) and (3). This profile shape is also consistent with the backplate temperature distributions reported by Wang et al.^30^ for similar unglazed absorber geometry.

### Economic evaluation

The economic analysis presented here provides a practical first-stage comparison between the UTSC and GTSC under the same local electricity tariff and operating assumptions. The thermal efficiencies reported in Sect. 3 are instantaneous steady-state values used to establish the operating behaviour of each collector under controlled boundary conditions, rather than year-round measured efficiencies.

The annual useful heat delivered reported in Table 4 was estimated by applying the experimentally validated one-dimensional analytical model to representative heating-season climatic inputs for Amman and summing the useful heat contribution over the relevant operating hours. The annual economic indicators should therefore be interpreted as model-based seasonal projections anchored to the measured steady-state dataset.

Table 4 summarises the economic parameters for both systems at the 6.0 m^2^ prototype scale, evaluated at the prevailing Jordanian electricity tariff of 0.10 USD/kWh. Capital costs cover absorber plate, framing, fan system, controller, instrumentation, and installation labour; the GTSC cost additionally includes the glass pane, aluminium spacers, and sealing.

To broaden the practical interpretation of the base-case result, an electricity-price sensitivity analysis is added in Table 5. Figure 5 presents the cost-benefit ratio CBR as a function of total installed area to illustrate the effect of scale.

**Table 4:** Economic parameters for the two prototype systems (A = 6.0 m², Amman, Jordan, 2025 tariff = 0.10 USD/kWh).

Economic metric	UTSC	GTSC
Capital cost [USD]	7,500	9,750
Annual useful heat delivered [kWh/year]	16,900	18,750
Annual energy saving [USD/year]	1,690	1,875
Simple payback period [years] (= 1/CBR)	4.4	5.2
Cost-benefit ratio CBR	0.225	0.192
Annual CO₂ reduction [kg/year] (gas @ 0.20 kg/kWh)	≈ 3,380	≈ 3,750

The UTSC achieves a higher CBR (0.225 vs. 0.192) and a shorter payback period (4.4 vs. 5.2 years), meaning it recovers a greater fraction of capital cost each year. However, the GTSC delivers higher absolute annual savings (1,875 vs. 1,690 USD/year), making it the more attractive option at larger installation scales where economies of scale in glazing, framing and ducting reduce the unit capital cost. Both payback periods are comfortably below the expected 20 + year service life of a galvanised steel absorber with protective coating.

**Table 5:** Sensitivity of annual energy savings and simple payback to electricity price.

Electricity price [USD/kWh]	UTSC annual saving [USD/year]	UTSC payback [years]	GTSC annual saving [USD/year]	GTSC payback [years]
0.08	1,352	5.55	1,500	6.50
0.10	1,690	4.44	1,875	5.20
0.12	2,028	3.70	2,250	4.33
0.15	2,535	2.96	2,813	3.47
0.20	3,380	2.22	3,750	2.60

As expected, higher electricity prices shorten the payback period for both systems. Within the analysed price range, the UTSC retains the shorter simple payback, whereas the GTSC provides the higher absolute annual savings.

### Multi-parameter performance analysis

**Fig. 16:**
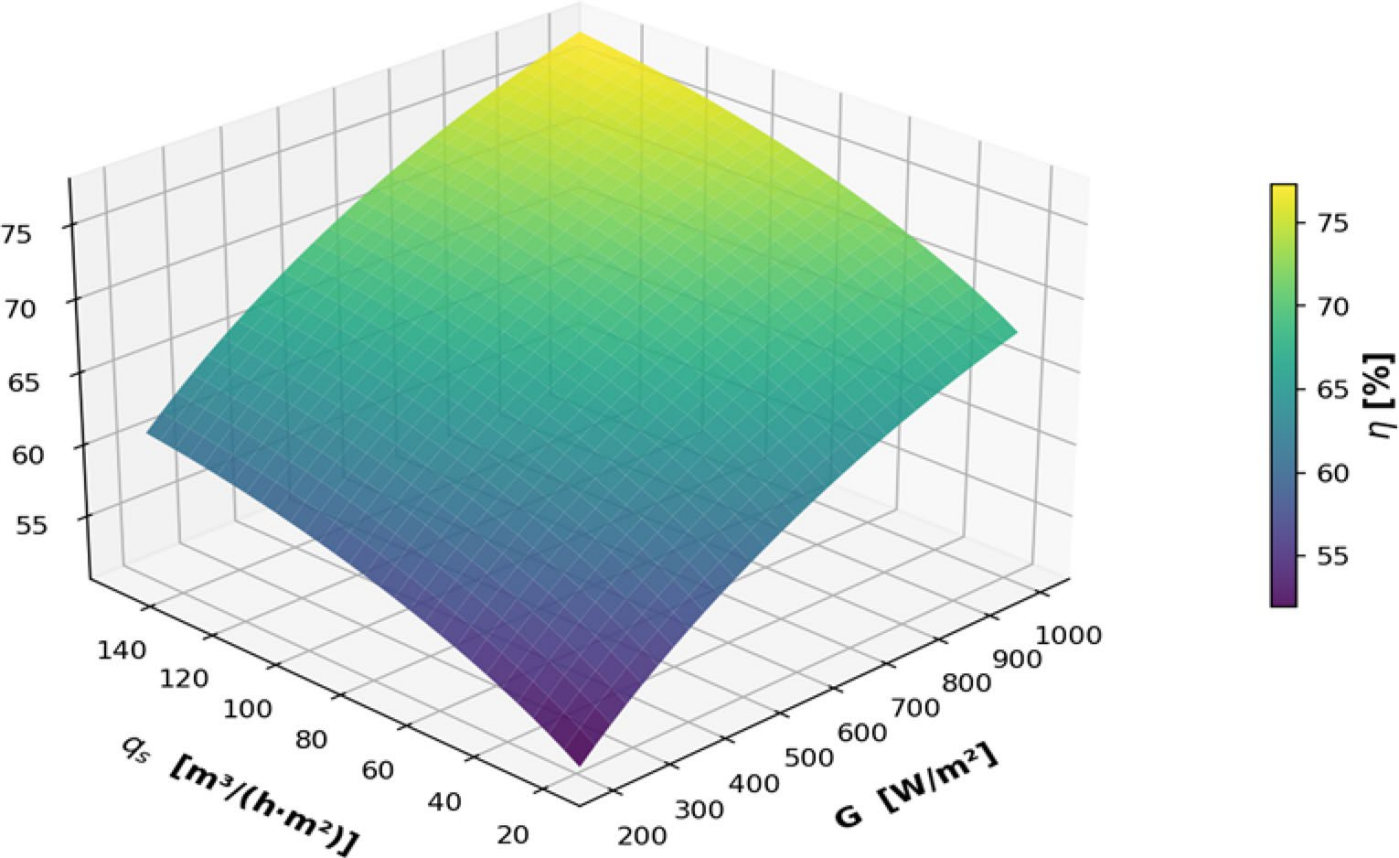
Analytical model surface: UTSC thermal efficiency η as a joint function of solar irradiance G and specific airflow rate q_s. Colour scale represents η [%].

Figure 16 shows the three-dimensional analytical model surface of UTSC thermal efficiency over the full G-q_s parameter space. A broad high-efficiency plateau (η > 60%) is visible at G > 600 W/m² combined with q_s > 72 m³/(h·m²). At low irradiance, radiative and convective losses dominate; at very low flow rate, the short air-plate contact time limits heat extraction. Fan control systems designed to operate within the plateau region will maximise annual energy yield while avoiding unnecessary electrical consumption.

**Fig. 17:**
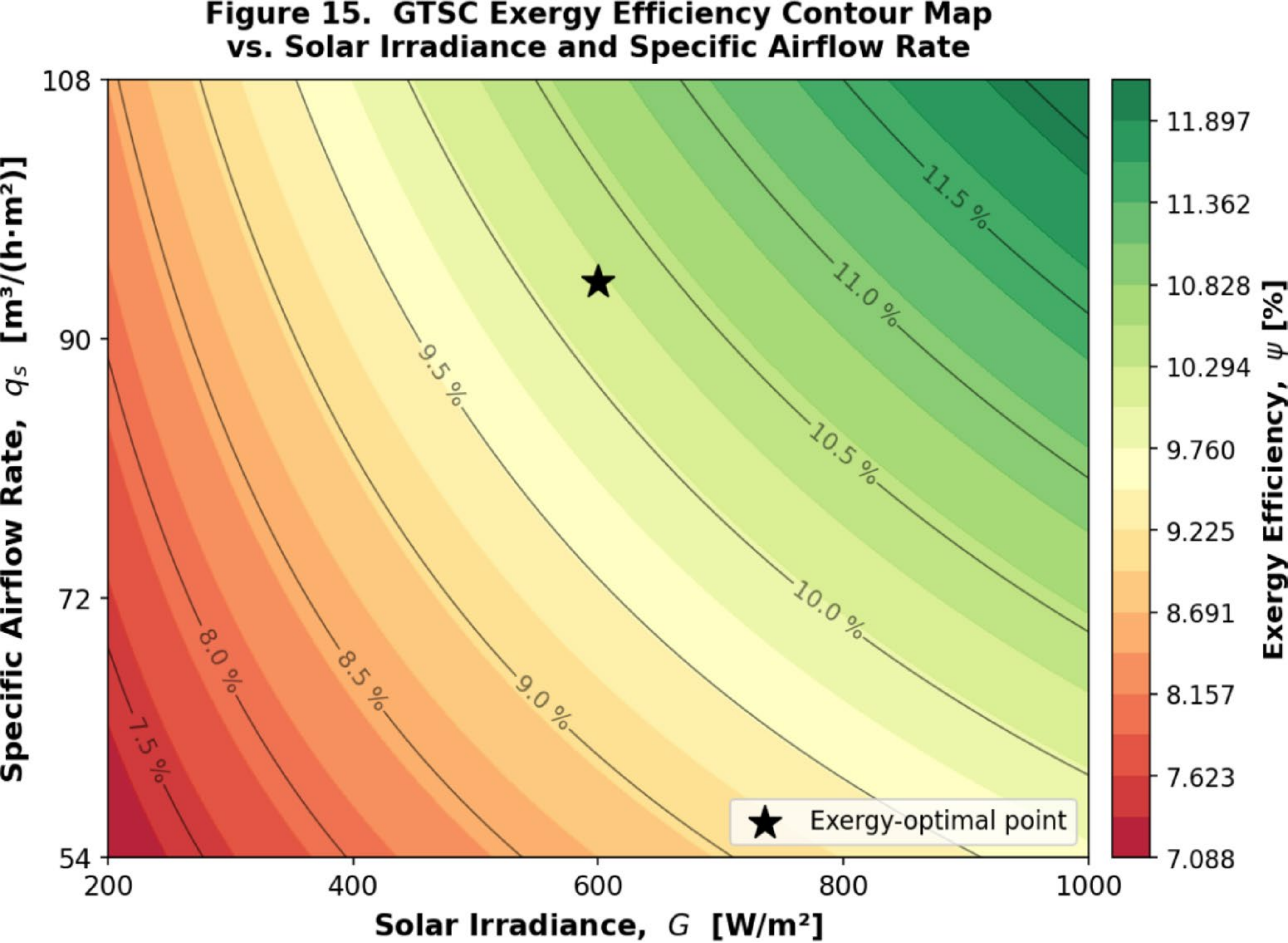
GTSC exergy efficiency ψ contour map as a function of G and q_s. ★: exergy-optimal operating point (G ≈ 600 W/m², q_s ≈ 94 m³/(h·m²)).

Figure 17 is a contour map of GTSC exergy efficiency ψ. Peak exergy efficiency (ψ ≈ 11%) occurs near G = 600 W/m² and q_s = 94 m³/(h·m²) (★). At higher irradiance, entropy generation during solar absorption grows faster than the exergy output. At lower flow rates, larger temperature gradients within the absorber increase local irreversibility. A smart-control algorithm targeting this operating point would simultaneously maximise thermodynamic efficiency and minimise supplementary heating demand.

### Energy balance breakdown

**Fig. 18:**
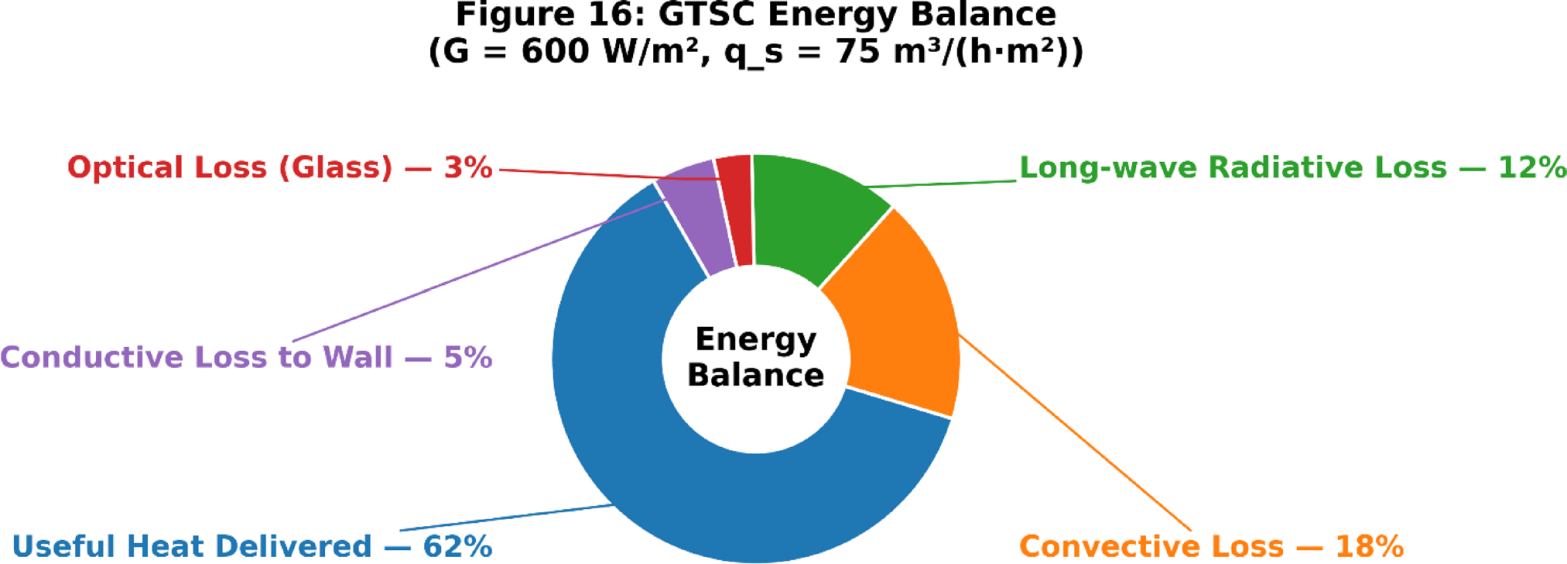
Analytical energy balance at G = 600 W/m² and q_s = 90 m³/(h·m²).

**Fig. 19:**
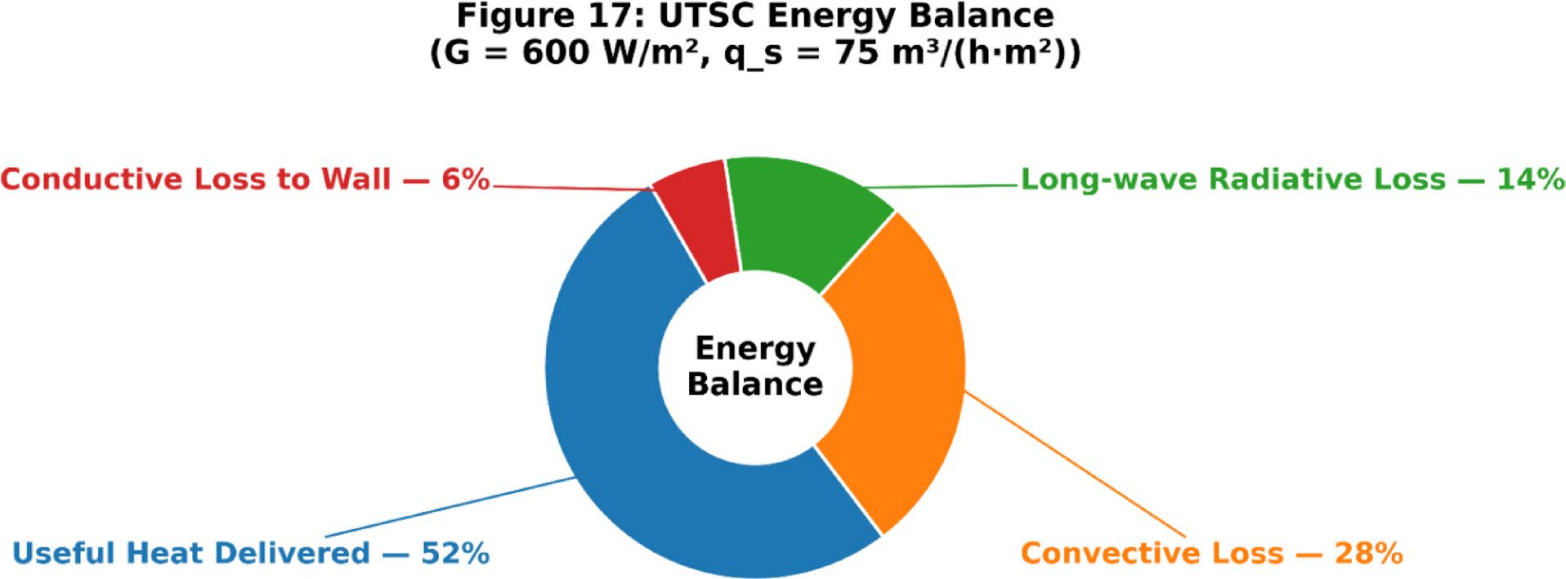
Analytical energy balance at G = 600 W/m² and q_s = 90 m³/(h·m²).

Figures 18 and 19 present the analytical energy balance for the GTSC and UTSC at G = 600 W/m² and q_s = 90 m³/(h·m²), partitioning absorbed solar energy among its main pathways. These distributions are derived from the validated analytical model, not from direct calorimetric measurement. For the GTSC, 62% of absorbed solar energy is delivered as useful heat; external convective loss from the glass outer surface accounts for 18%; long-wave radiative emission from the glass for 12%; conductive loss through the back wall for 5%; and optical absorption/reflection at the glass surface for the remaining 3%. For the UTSC, the absence of a glass cover raises convective loss to 28% and long-wave radiative emission from the directly exposed hot absorber to 14%, while eliminating the optical penalty; useful heat delivery falls to 52%. The net effect of glazing is to convert 10 additional percentage points of convective loss and 2% points of radiative loss into useful heat, at the cost of 3% optical loss - a net gain of 7% points in useful heat fraction, consistent with the measured η difference in Fig. 3.

Figure 18 shows the GTSC energy balance, with 62% useful heat delivered.

Figure 19 shows the UTSC energy balance, with 52% useful heat delivered. Adding glazing reduces convective and radiative losses while introducing a modest optical penalty.

## Discussion

### Thermal performance in context

The measured UTSC efficiencies of 42–65% fall within the expected range for outdoor unglazed flat-plate air heaters reported in the literature. Collins and Abulkhair^37^ measured heat exchange effectiveness values of 0.7–0.9 for similar unglazed geometries, consistent with the ε values of 0.77–0.91 measured here. The GTSC efficiencies of 48–75% match the results of Mahmood^25^(48–75% for a glazed flat-plate air heater under comparable semi-arid conditions) and are within the improvement range reported by Gao et al.^23^ relative to their unglazed reference.

The consistent 6–9% point advantage of the GTSC arises from two complementary mechanisms. First, at low irradiance, the absorber temperature is only modestly above ambient, and convective heat loss from the unglazed surface is proportionally large; glazing nearly eliminates this loss. Second, at high irradiance, the absorber is significantly hotter and long-wave radiative emission from the black plate to the cold sky becomes the dominant loss pathway; the glass cover acts as a long-wave radiative trap. These two effects together produce an advantage that varies only slightly across the irradiance range, which is valuable for building applications that operate under widely varying solar conditions throughout a heating season.

### Implications for NZEB-oriented design and climatic transferability

The present findings are relevant to NZEB-oriented building design because both tested systems reduce the auxiliary heating demand associated with ventilation air, while the UTSC also reduces conductive heat loss through dynamic-insulation action. In climates with good winter solar availability and moderate daytime ambient temperatures, such as semi-arid Mediterranean regions, the GTSC is attractive where stable ventilation preheating and reduced sensitivity to external convective loss are prioritised.

In colder climates, the benefit of glazing may become more important because reductions in radiative and convective losses become increasingly valuable as the absorber-to-ambient temperature difference increases. In windier climates, the relative advantage of glazing may also increase, although the present study does not quantify that effect because the experiments were intentionally restricted to V ∞ ≤ 2 m/s. In milder climates or cost-sensitive retrofits, the UTSC may remain attractive because of its shorter payback period and its strong wall heat-loss recapture behaviour under lower-irradiance winter conditions. The results therefore support climate-responsive selection of TSC configuration rather than a single universal preference.

### Dynamic insulation: a complementary perspective

The RI and VLR results describe two complementary collector-boundary effects. RI represents the reduction in conductive wall heat loss through the UTSC wall section, while VLR represents the calculated reduction in supplementary heating required for the GTSC outlet air stream to reach the reference supply-air set-point. Therefore, these two metrics should be interpreted as distinct performance indicators rather than as direct measures of the same room-level thermal response.

The UTSC showed its strongest wall heat-loss recapture under lower irradiance conditions, when outward conductive wall heat loss remained important and the suction flow could intercept part of this heat transfer through the wall section. In contrast, the GTSC showed its strongest ventilation-air preheating benefit under higher irradiance conditions, when the collector outlet temperature increased and the calculated residual supply-air heating requirement decreased. This behaviour confirms the complementary role of the two configurations: the UTSC is more effective for dynamic-insulation benefit, whereas the GTSC is more effective for raising the temperature of the delivered ventilation air.

### Exergy perspective

The low absolute exergy efficiencies (ψ < 12%) reflect a general limitation of solar air heaters rather than a deficiency specific to this design: converting high-quality solar exergy (≅ 4333 K) to warm air at 30–45 °C involves large thermodynamic irreversibility regardless of collector type^34–36^. The low absolute values, well below 15%, reflect the thermodynamic limitation common to solar air heaters: solar exergy enters at a very high source temperature, whereas the useful output is delivered as warm air only slightly above ambient, which results in unavoidable entropy generation during the conversion process.

The practical implication is that the exergy-optimal operating point identified in Fig. 17 (★, G ≈ 600 W/m², q_s ≈ 94 m³/(h·m²)) should be viewed as a guide for smart-control strategies that schedule TSC operation to coincide with moderate irradiance and run fans near the 90 m³/(h·m²) plateau in order to simultaneously maximise both thermal and exergetic output. At very high irradiance (η continues to rise but ψ plateaus or declines), it may be preferable to increase flow rate further to extract more heat, accepting a marginal reduction in thermodynamic quality but avoiding absorber overheating.

### Natural convection for passive integration

The natural-convection results (Fig. 10) demonstrate that 55% thermal efficiency is achievable at H = 3.0 m without any fan power - a result made possible by the glass cover retaining heat within the cavity and maintaining the buoyancy driving force. This performance level is practically significant: it is comparable to the forced-convection efficiency of the UTSC at the lowest tested flow rate and sufficient to meaningfully reduce ventilation preheating demand in single-storey residential buildings. From an energy perspective, eliminating the fan system removes the largest non-solar operating cost of the installation; from a maintenance perspective, it removes the primary mechanical failure mode. The minimum cavity height of 2.5 m required to achieve η > 40% in passive mode is realisable on most single-storey buildings.

From a façade-design perspective, these passive results indicate that cavity height is one of the governing parameters for fan-free GTSC operation. For the tested geometry, passive performance becomes clearly more viable once the effective height approaches 2.5 to 3.0 m, because the buoyancy pressure difference becomes sufficient to sustain a meaningful airflow rate and temperature rise.

This makes the concept particularly relevant to single-storey façades, stairwells, atrium-side walls, and other vertically continuous façade zones where uninterrupted cavity height can be preserved. At the same time, the present results should not be interpreted as a direct design rule for all multi-storey façades, because taller systems may experience additional flow non-uniformity, façade leakage, structural constraints, and integration issues that were outside the scope of the present study. The passive dataset should therefore be read as a building-scale proof of feasibility for compact glazed TSC integration rather than as a complete design chart for tall façades.

### Design recommendations

Drawing together the results from Figs. 3, 4, 5, 6, 7, 8, 9, 10, 11, 12, 13, 14, 15, 16, 17 and 18, the following design guidelines are proposed for TSC systems in Amman and climatically comparable semi-arid Mediterranean environments:

- Perforation geometry. The validated 1 mm hole diameter at 15 mm pitch (nominal porosity 0.3%) delivers ε > 0.70 across all tested flow rates with the 110 mm plenum. Increasing porosity beyond 1% would reduce flow resistance but risk non-uniform suction distribution, as noted by Leon and Kumar^16^.
- Glass cover gap. The 30 mm glass-to-absorber distance is near-optimal for suppressing internal convective loss without overheating. Low-iron glass (high solar transmittance τ = 0.91) is recommended to minimise the optical penalty; standard float glass with τ ≈ 0.82 would eliminate 2–3% points of efficiency advantage.
- Operating flow rate. A specific airflow rate of 72–90 m³/(h·m²) represents the best operating zone for combined thermal efficiency and useful temperature rise. This corresponds to approximately 40–50% of the rated fan capacity in this study and is well within the low-noise operating range of the fans. In forced-convection operation, fan-assisted airflow should be disabled when useful solar preheating is unavailable, so that non-solar periods do not introduce avoidable parasitic heat loss.
- Cavity height for passive operation. GTSC passive efficiency exceeds 40% only for H ≥ 2.5 m. For multi-storey applications with H > 3.0 m, further efficiency gains can be expected beyond the range tested here.
- System selection criteria. The UTSC is preferred for dynamic-insulation priority, cost-constrained projects, or simple roof-mounted air preheaters. The GTSC is preferred for projects requiring maximum absolute energy delivery, passive natural-convection operation, or consistent efficiency under variable or moderate irradiance conditions.

### Limitations and future research

The experimental programme was conducted as a controlled comparative study under calm-weather, quasi-steady conditions rather than as a full all-weather performance campaign. Tests were restricted to clear-sky periods with V ∞ ≤ 2 m/s so that the glazing effect could be isolated under matched boundary conditions. The present study reports collector-boundary performance and wall-flux-based metrics; it does not constitute a full occupied-room thermal-comfort assessment.

This approach was appropriate for the comparative objective of the study, but it does not quantify the behaviour of the UTSC and GTSC under stronger winds, wind gusts, or rapidly fluctuating irradiance. Under such conditions, the relative advantage of the GTSC may change because the external convective penalty on the UTSC is expected to become more pronounced^21,22^. Quantifying that effect requires a dedicated transient and higher-wind experimental programme.

The present dataset covers daytime solar operation only. Zero-irradiance and night-time conditions were not evaluated experimentally. In practical operation, forced airflow through the collector should be disabled when useful solar preheating is unavailable, unless a separate night-time operating strategy is specifically being investigated. This avoids avoidable parasitic heat loss during non-solar periods and keeps the interpretation of the present results consistent with solar-assisted daytime operation. The current annual energy estimate should therefore be interpreted as solar-assisted daytime performance only and not as a quantified assessment of nocturnal collector behaviour.

Prior work by the authors has already examined TSC heat-flux behaviour, wall heat-loss recapture, and dynamic-insulation operation beyond the immediate daytime heating period^28,32^. However, the present study does not provide a dedicated paired overnight comparison between the GTSC and the UTSC under matched indoor and ambient conditions. Such a comparison would be a valuable extension of the present work for quantifying post-sunset heat retention and directly comparing nocturnal behaviour between glazed and unglazed configurations.

Extended monitoring of both configurations under actual service conditions would refine the economic comparison further by quantifying long-term performance evolution, maintenance requirements, and reliability effects.

The one-dimensional analytical model was appropriate for the comparative objectives of this study but does not resolve the three-dimensional velocity and temperature fields within the glass-to-absorber cavity. CFD simulation of the GTSC configuration would provide spatial detail on the internal heat-transfer pathways and cavity flow structure that lies beyond the resolution of the present thermocouple arrangement, and would offer a rigorous basis for further model refinement.

## Conclusions

A systematic side-by-side experimental and analytical study of glazed and unglazed transpired solar collectors sharing an identical absorber plate was carried out on a south-facing building façade in Amman, Jordan. Both modules were tested simultaneously under clear-sky winter conditions over specific airflow rates of 18–144 m^3^/(h· m^2^) and solar irradiance levels of 200–1000 W/m^2^. Six performance indices, namely thermal efficiency, heat exchange effectiveness, exergy efficiency, wall heat-loss recapture index, ventilation load reduction, and cost-benefit ratio, were evaluated using physically consistent definitions that allow direct comparison with other studies. The principal findings are as follows.

1. Glazing improved thermal performance under the investigated conditions. The GTSC achieved thermal efficiencies of 48–75% and exergy efficiencies up to 12%, while the identical-geometry UTSC achieved 42–65%, giving an advantage of 6–9% points across the tested irradiance levels (200–1000 W/m^2^) and specific airflow rates (18–144 m^3^/(h·m^2^)). These results indicate that the GTSC offers improved performance within the examined operating range, particularly under variable solar conditions.
2. Dynamic insulation and ventilation-air preheating showed complementary collector-boundary behaviour. The UTSC wall heat-loss recapture index RI decreased monotonically from 93% at low irradiance to 63% at peak irradiance, indicating that wall heat-loss recapture was strongest under lower irradiance conditions with outward reference-wall heat flux. The GTSC ventilation load reduction VLR increased monotonically from 60% to 90% with irradiance, representing the calculated reduction in supplementary heating required for the outlet air stream to reach the reference supply-air set-point. Both metrics remained within the physically meaningful range of (0% − 100%) under the definitions adopted in this study.
3. Passive natural-convection operation was shown to be feasible for the tested GTSC configuration. The GTSC cavity driven by buoyancy alone achieved η = 55% at H = 3.0 m without fan energy, which is comparable to forced-convection performance at the lowest tested flow rate. For the tested geometry and conditions, cavity heights of at least 2.5 m were associated with efficiencies above 40% in passive mode.
4. The energy-balance analysis supported the thermal role of glazing. At G = 600 W/m^2^ and q_s = 90 m^3^/(h·m^2^), the GTSC converted 62% of absorbed solar energy to useful heat, compared with 52% for the UTSC. Under the same condition, adding a glass cover reduced convective loss from 28% to 18% and radiative loss from 14% to 12%, with an optical penalty of 3%, yielding a net improvement of 7% points.
5. Both systems showed promising economic performance within the assumptions adopted in this study. The simple payback periods were estimated as 4.4 years for the UTSC and 5.2 years for the GTSC in the Jordanian energy market at 0.10 USD/kWh. Under these assumptions, the UTSC recovered the initial cost more rapidly, whereas the GTSC provided greater annual savings. Annual CO₂ reductions of approximately 3,000 kg or more per 6 m² installation also indicate environmental benefit.

The validated model and design charts are ready for practical application. The one-dimensional analytical model, validated to within ± 10% against measurements, together with the three-dimensional efficiency surface (Fig. 16), exergy contour map (Fig. 17), and energy-balance pie charts (Figs. 18 and 19), offers practical guidance for TSC assessment and optimisation. However, these tools should be applied with appropriate caution outside the directly tested parameter range, and further validation under additional climatic conditions and system configurations remains necessary.

## Data Availability

The datasets generated and/or analysed during the current study are not publicly available due to institutional data management restrictions, but are available from the corresponding author on reasonable request.

## Abbreviations

CBR: Cost-benefit ratio (-)
CFD: Computational fluid dynamics (-)
GTSC: Glazed transpired solar collector (-)
HVAC: Heating, ventilation and air-conditioning (-)
NC: Natural convection (-)
NZEB: Nearly zero-energy building (-)
RI: Wall heat-loss recapture index (-)
RSS: Root-sum-of-squares (-)
TSC: Transpired solar collector (-)
UTSC: Unglazed transpired solar collector (-)
XPS: Extruded polystyrene insulation board (-)

## Latin symbols

A: Effective collector area (m^2^)
c_p_: Specific heat of air at constant pressure (J/(kg K) )
CBR: Cost-benefit ratio (annual saving [USD] / capital cost [USD] (-))
d: Perforation hole diameter (mm)
G: Solar irradiance on the façade plane (W/m^2^)
g: Gravitational acceleration (m/s^2^)
H: Cavity height (m)
H_ref: Reference cavity height (m)
h_c_: Convective heat-transfer coefficient: W/(m^2^ K)
k: Thermal conductivity (W/(m K))
p: Perforation pitch centre-to-centre (mm)
P_heat: Supplementary heating power (W)
q_cond: Conductive heat flux through wall (W/m^2^)
Q_u: Useful heat gain (W)
q_s: Specific volumetric airflow rate (m^3^/(h m^2^))
Re: Reynolds number (-)
RI: Wall heat-loss recapture index (%)
T: Temperature (°C or K)
t: Absorber plate thickness (mm)
V̇: Volumetric flow rate produced by the fan (m^3^/h)
VLR: Ventilation load reduction (%)

## Greek symbols

α: Solar absorptance of the absorber coating (-)
ΔP: Buoyancy pressure difference (N/m^2^)
ΔT: Outlet temperature rise (°C)
ε: Heat exchange effectiveness (-)
ε_g_: Long-wave thermal emittance of glass cover(-)
ε_p_: Long-wave thermal emittance of absorber coating (-)
η: Thermal efficiency (%)
µ: Dynamic viscosity of air (kg/(m s))
ρ: Air density (kg/m^3^)
τ: Solar transmittance of glass cover (-)
ϕ: Porosity of absorber plate (%)
ψ: Exergy efficiency (%)
δ: Thickness: m

## Subscripts

amb: Ambient air (-)
c: Cavity (glass-to-absorber space, GTSC only) (-)
cond: Conductive (-)
in: Air inlet (-)
out: Air outlet (-)
p: Absorber/perforated steel plate (-)
ref: Reference wall section (-)
sp: Indoor temperature set-point (-)
sun: Effective black-body temperature of solar radiation (-)
w: wall (-)

## References

[1] Heyhat, M., Mahdi, A. I., Al-Shiblawi, O., Haghani & Mostafa Bahmani. Hydrophobic wavy tape to augment the parabolic trough solar collector performance. *Sci. Rep.* **15** (1), 36231 (2025). 41102422 10.1038/s41598-025-20173-9 PMC12533242

[2] Heyhat, M., Mahdi, M. Q. J. & Abbood Jabraeil Ahbabi Saray, and Abolghasem Mokhtari Ardekani. Comparative assessment of direct absorption solar collector performance in different climates. *Sci. Rep.* **13** (1), 21359 (2023). 38049476 10.1038/s41598-023-48780-4 PMC10695963

[3] Donga, R. K., Kumar, S. & Velidi, G. Numerical investigation of performance and exergy analysis in parabolic trough solar collectors. *Sci. Rep.* **14** (1), 31908 (2024). 39738406 10.1038/s41598-024-83219-4 PMC11685669

[4] Ben Khedher, N. et al. Variable viscosity and activation energy aspects in convection heat transfer over gravity driven solar collector plate for thermal energy storage. *Sci. Rep.* **14** (1), 27239 (2024). 39516555 10.1038/s41598-024-77715-w PMC11549296

[5] Amar, M. et al. Nabisab Mujawar Mubarak, and Md Abul Kalam. Energy, exergy and economic (3E) analysis of flat-plate solar collector using novel environmental friendly nanofluid. *Sci. Rep.* **13** (1), 411 (2023). 36624198 10.1038/s41598-023-27491-w PMC9829679

[6] Ma’in, F. Enhancing sustainable hospital design in Jordan: An AHP-based approach to energy efficiency and environmental protection. *Results Eng.* **26**, 105319 (2025).

[7] Al-Shalabi, M., Shehab, M., Alshammari, M. T., Alazmi, M. & Alrawashdeh, R. O. Laith Abualigah, and Mohammed A. Mahdi. Optimal sizing of smart hybrid renewable energy system using Lotus Effect Optimization Algorithm. *Energy Rep.* **14**, 1936–1948 (2025).

[8] Emamjome Kashan, M., Fung, A. S. & Eisapour, A. H. Insulated concrete form foundation wall as solar thermal energy storage for cold-climate building heating. *Energy Convers. Management: X*. **19**, 100391 (2023).

[9] Akhan, H. & Eryener, D. Building integrated solar air heating with waste heat utilization. *Energy. Conv. Manag.* **157**, 136–145 (2018).

[10] Román, F. & Hensel, O. Comparison of steady-state and transient modelling for solar air heater performance prediction. *Energy Convers. Management: X*. **16**, 100327 (2022).

[11] Fawaier, M. & Bokor, B. Dynamic insulation systems of building envelopes: a review. *Energy Build.* **270**, 112268 (2022).

[12] Bokor, B. & Kajtár, L. Transpired solar collectors in building service engineering: combined operation and special applications. *Int. Rev. Appl. Sci. Eng.* **9**, 65–71 (2018).

[13] Kutscher, C. F., Christensen, C. B. & Barker, G. M. Unglazed transpired solar collectors: heat loss theory. *J. Sol. Energy Eng.* **115**, 182–188 (1993).

[14] Kutscher, C. F. Heat exchange effectiveness and pressure drop for air flow through perforated plates with and without crosswind. *J. Heat Transfer*. **116**, 391–399 (1994).

[15] Van Decker, G. W. E., Hollands, K. G. T. & Brunger, A. P. Heat-exchange relations for unglazed transpired solar collectors with circular holes on square or triangular pitch. *Sol. Energy*. **71**, 33–45 (2001).

[16] Leon, M. A. & Kumar, S. Mathematical modelling and thermal performance analysis of unglazed transpired solar collectors. *Sol. Energy*. **81**, 62–75 (2007).

[17] Peci, F., Táboas, F., Ruiz de Adana, M. & Comino, F. Experimental study of overheating of an unglazed transpired collector façade under southern European summer conditions. *Sol. Energy*. **189**, 194–206 (2019).

[18] Wang, D. et al. Experimental study on heating characteristics and parameter optimisation of transpired solar collectors. *Appl. Energy*. **238**, 534–546 (2019).

[19] Fawaier, M., Bokor, B. & Horváth, M. Wall heat loss recapture evaluation of transpired solar collectors for different European climates. *Case Stud. Therm. Eng.* **24**, 100836 (2021).

[20] Badescu, V. et al. Regularizing operation of unglazed transpired collectors by incorporating phase change materials. *Energy. Conv. Manag.* **184**, 681–708 (2019).

[21] Gawlik, K. M. & Kutscher, C. F. Wind heat loss from corrugated, transpired solar collectors. *J. Sol. Energy Eng.* **124**, 256–261 (2002).

[22] Bandara, W., Amarasekara, B. K. & Rupasinghe, C. P. Comparative assessment of efficiency of unglazed and glazed transpired solar collectors. *Procedia Eng.* **212**, 1295–1302 (2018).

[23] Gao, M., Wang, D., Liu, Y., Wang, Y. & Zhou, Y. Thermal performance of a novel glazed transpired solar collector with perforating corrugated plate. *J. Clean. Prod.* **257**, 120443 (2020).

[24] Gao, M., Fan, J., Furbo, S. & Xiang, Y. Energy and exergy analysis of a glazed solar preheating collector wall. *Renew. Energy*. **196**, 1048–1063 (2022).

[25] Mahmood, A. J. Experimental investigation of thermal efficiency and economic feasibility of glazed and unglazed solar air heaters. *Results Eng.* 106004. (2025).

[26] Alrwashdeh, S., Alsaraireh, F. M. & Saraireh, M. A. Solar radiation map of Jordan governorates. *Int. J. Eng. Technol.* **7**, 1664–1667 (2018).

[27] Al-Kloub, M. M. et al. *Model simulations of local meteorological conditions in Jordan* (Jordan Journal of Earth and Environmental Sciences, 2020).

[28] Fawaier, M., Abu Shaban, N. & Bokor, B. Heat transfer investigation for wall heat loss recapture in transpired solar collectors. *Energy Convers. Management: X*. **22**, 100540 (2024).

[29] Hollick, J. C. Unglazed solar wall air heaters. *Renew. Energy*. **5**, 415–421 (1994).

[30] Wang, D. et al. Experimental and numerical study of airflow and thermal characteristics of a non-uniform transpired solar collector. *Build. Simul.* **13**, 1305–1319 (2020).

[31] Kutscher, C., Christensen, C. & Gawlik, K. Letter to the editor. *Sol. Energy*. **74** (4), 353–354 (2003).

[32] Fawaier, M., Róbert, G. & Bokor, B. Simulation study of dynamic building insulation with transpired solar collectors. *Energy Reports* **12**, 1325–1343 (2024).

[33] Eryener, D., Hollick, J. & Kuscu, H. Thermal performance of a transpired solar collector updraft tower. *Energy. Conv. Manag.* **142**, 286–295 (2017).

[34] Petela, REoutrS. Exergy of undiluted thermal radiation. *Solar Energy* **74**, 469–488 (2003).

[35] Petela, R. Exergy of heat radiation. *J. Heat Transfer* **86**, 187–192 (1964).

[36] Nazari, M., Jafarmadar, S. & Khalilarya, S. Exergy and thermoeconomic analyses of serpentine tube flat-plate solar water heaters coated with CuO nanostructures, Case Stud. *Therm. Eng.* **35**, 102072 (2022).

[37] Collins, M. R. & Abulkhair, H. Evaluation of heat transfer and effectiveness for unglazed transpired solar air heaters. *Sol. Energy*. **99**, 231–245 (2014).

